# The genomic signature of wild‐to‐crop introgression during the domestication of scarlet runner bean (*Phaseolus coccineus* L.)

**DOI:** 10.1002/evl3.285

**Published:** 2022-06-15

**Authors:** Azalea Guerra‐García, Idalia C. Rojas‐Barrera, Jeffrey Ross‐Ibarra, Roberto Papa, Daniel Piñero

**Affiliations:** ^1^ Departamento de Ecología Evolutiva, Instituto de Ecología Universidad Nacional Autónoma de México Ciudad de México 04510 México; ^2^ Department of Plant Sciences University of Saskatchewan Saskatoon SK S7N 5A2 Canada; ^3^ Environmental Genomics Max Planck Institute for Evolutionary Biology 24306 Plön Germany; ^4^ Department of Evolution and Ecology, Center for Population Biology, and Genome Center University of California, Davis Davis California 95616; ^5^ Dipartimento di Scienze Agrarie, Alimentari ed Ambientali Università Politecnica delle Marche Ancona 60131 Italy

**Keywords:** Domestication, gene flow, introgression, legumes, population genomics, wild relatives

## Abstract

The scarlet runner bean (*Phaseolus coccineus*) is one of the five domesticated *Phaseolus* species. It is cultivated in small‐scale agriculture in the highlands of Mesoamerica for its dry seeds and immature pods, and unlike the other domesticated beans, *P. coccineus* is an open‐pollinated legume. Contrasting with its close relative, the common bean, few studies focusing on its domestication history have been conducted. Demographic bottlenecks associated with domestication might reduce genetic diversity and facilitate the accumulation of deleterious mutations. Conversely, introgression from wild relatives could be a source of variation. Using Genotyping by Sequencing data (79,286 single‐nucleotide variants) from 237 cultivated and wild samples, we evaluated the demographic history of traditional varieties from different regions of Mexico and looked for evidence of introgression between sympatric wild and cultivated populations. Traditional varieties have high levels of diversity, even though there is evidence of a severe initial genetic bottleneck followed by a population expansion. Introgression from wild to domesticated populations was detected, which might contribute to the recovery of the genetic variation. Introgression has occurred at different times: constantly in the center of Mexico; recently in the North West; and anciently in the South. Several factors are acting together to increase and maintain genetic diversity in *P. coccineus* cultivars, such as demographic expansion and introgression. Wild relatives represent a valuable genetic resource and have played a key role in scarlet runner bean evolution via introgression into traditional varieties.

The scarlet runner bean (*Phaseolus coccineus* L.) is one of the five domesticated *Phaseolus* species. It is a close relative of common bean (*Phaseolus vulgaris*) and year bean (*Phaseolus dumosus*), which were also domesticated in Mesoamerica. In contrast with the common bean, an autogamous annual species, the scarlet runner bean is allogamous and perennial. It inhabits the highlands of Mesoamerica (1000–3000 m.a.s.l.), from northern Mexico (Chihuahua) to Panama (Delgado‐Salinas 1988). It is usually cultivated as an annual crop for dry seed and immature pods. Because of the high phenotypic variation of runner bean, Freytag and Debouck (2002) proposed two subspecies: *P. coccineus* subsp. *coccineus*, a red‐flowered type including 11 varieties (one of these is the cultivated form), and *P. coccineus* subsp. *striatus*, a purple‐flowered type with eight wild varieties.

The domesticated form of *P. coccineus* is cultivated in Mexico, Guatemala, and Honduras (Delgado‐Salinas 1988); due to its tolerance to cold, it is also cultivated in European countries such as the United Kingdom, Netherlands, Italy, and Spain (Rodiño et al. 2007).

Two domestication events for *P. coccineus* were initially suggested using low‐resolution molecular markers and a focus on European cultivars (Spataro et al. 2011; Rodriguez et al. 2013). More recently Guerra‐García et al. (2017) proposed a single domestication event, which probably took place in the central Mexican biogeographic area known as the Trans Mexican Volcanic Belt (TMVB).

The demographic history of crops shapes patterns and levels of genetic variation on which natural and artificial selection can act (Meyer and Purugganan 2013; Gaut et al. 2018). The first stages of domestication are often associated with genetic bottlenecks because early farmers likely interacted with a subset of wild populations for initial management (Meyer and Purugganan 2013; Gaut et al. 2018). Furthermore, population size changes and gene flow between the wild relatives and the incipient crops play a role in determining levels of genetic variation. The subsequent range expansion out of the center of origin leads to the adaptation of the domesticated species to different environments as well as distinct cultural preferences (Meyer and Purugganan 2013; Gaut et al. 2015; Janzen et al. 2019). Hybridization during domestication has been widely documented (Stewart et al. 2003; Arnold 2004; Hancock 2012; Bredeson et al. 2016; Choi and Purugganan 2018) and evidence suggests that wild‐to‐crop introgression and even interspecific hybridization can be a source of adaptive variation (Janzen et al. 2019; Purugganan 2019). One example is the case of maize adaptation to highlands as a result of introgression from wild populations of *Zea mays* ssp. *mexicana* (van Heerwaarden et al. 2011; Hufford et al. 2013; Takuno et al. 2015).

The demographic bottlenecks associated with domestication might also lead to a reduction in the effectiveness of selection (Morrell et al. 2012; Moyers et al. 2018). The increased genetic load in crops is called the “cost of domestication” and it has been documented in species like rice (Lu et al. 2006), maize (Mezmouk and Ross‐Ibarra 2014), sunflower (Renaut and Rieseberg 2015), and cassava (Ramu et al. 2017).

In this work, we investigate the demographic history of scarlet runner bean during its domestication and subsequent spread, the role of gene flow between wild and domesticated populations, and how these processes have shaped the genetic diversity present in the populations of *P. coccineus* in Mexico.

## Methods

### SAMPLING AND GENOMIC DATA

Plant material was collected from Northwest (Durango) to Southeast of Mexico (Chiapas) during 2014 and 2015. Wild individuals were sampled in nine locations, ferals in two sites, and traditional varieties at 11 locations. Samples of the breeding line Blanco Tlaxcala and a cultivar from Spain were also included (Table S1). Categories (wild, feral, traditional variety) were assigned according to habitat and morphological observations. One of the wild populations corresponded to subsp. *striatus*. Samples from *P. vulgaris* and *P. dumosus* were included and used as outgroups.

Leaf tissue from wild samples was collected and stored in silica until processed. Seeds from traditional varieties were germinated and DNA was extracted using a DNeasy Plant Mini Kit (Qiagen). Library preparation and sequencing were performed at the Institute for Genomic Diversity at Cornell University. For library construction, a double digestion was performed using PstI and BfaI enzymes, following Genotyping by Sequencing protocol (Elshire et al. 2011). A total of 326 samples were sequenced in four lanes of an Illumina HiSeq 2500 (100 bp, single‐end reads).

### VARIANT DISCOVERY AND FILTERING

Fastq files were demultiplexed with GBSx 1.3 (Herten et al. 2015) and reads were trimmed with Trimmomatic 0.36 (Bolger et al. 2014). Alignments were performed with Nextgenmap 0.5.3 (Sedlazeck et al. 2013) using the *Phaseolus vulgaris* genome version 2.1 (DOE‐JGI and USDA‐NIFA, http://phytozome.jgi.doe.gov/) and then were converted to binary files using samtools 1.5 (Kaisers et al. 2015). Single‐nucleotide variants (SNVs) were discovered for each sample using the HaplotypeCaller tool and genotypes were then merged with GenotypeGVCFs. Both tools are from the Genome Analysis Toolkit (GATK 4.0.1.0; McKenna et al. 2010).

VCFtools 0.1.15 (Danecek et al. 2011) was used to perform the variant filtering according to the following parameters: minimum mean depth 6×; max missingness per sample 0.30; max missingness per site 0.05; loci not mapped in *P. vulgaris* chromosomes were excluded; and only biallelic sites were kept. SNVs that were not in Hardy‐Weinberg equilibrium (*P* < 0.01) in at least one wild population were identified with PLINK 1.07 (Chang et al. 2015) and filtered, as well as the 15,601 putative paralogs detected with HDplot (McKinney et al. 2017).

Because the number of samples per population significantly varies (see *Results*), a second filtering was applied to reduce the difference in the sampling. For this filtering, relatedness was estimated through VCFtools 0.1.15 (Danecek et al. 2011), using the relatedness2 function, based on the method of Manichaikul et al. (2010). When a pair of individuals from the same population presented a relatedness >0.05, one of the samples was excluded. Individuals with a missingness >0.15 were also removed.

We classified the SNVs into three categories: nongenic, intronic, and coding regions (CDS). The consequence of SNVs within coding regions was predicted with the R package VariantAnnotation (Obenchain et al. 2014).

### DEFINING “POPULATIONS”

Diversity analyses were performed at the “population” level. Populations were established according to (1) Principal Component Analysis (PCA) performed with SNPrelate (Zheng et al. 2012); (2) the genetic groups identified with Admixture version 1.3 (Alexander et al. 2009); and (3) the topology of the phylogenetic hypothesis constructed with FastTree (Price et al. 2009). Populations may differ from locations because in some cases individuals from different locations belonged to the same genetic group. In other cases, genetic groups were split because a clear differentiation was observed in the PCA and in the phylogenetic tree. The nature of the samples was also considered (e.g., feral, breeding line, or traditional variety).

### MEASURING DIVERSITY

Heterozygosity and inbreeding coefficient (*F*_IS_) per site were estimated with the Hierfstat package (Goudet 2005), performing a bootstrap (1000) to obtain confidence intervals for the inbreeding coefficient, and Kruskal‐Wallis and Pairwise test to compare the heterozygosity among populations. Hierfstat was also used to obtain the differentiation index (pairwise *F*_ST_) among the established populations according to Weir and Cockerham (1984). Genetic diversity was also estimated with the data subset, which had a lower variation in sample size.

A custom R script was made to discover the private SNVs within each population, considering only the polymorphic sites within the groups. This R script uses the Hierfstat package (Goudet 2005) to estimate allele frequencies. We applied a rarefaction approach for allelic richness and private allelic richness using ADZE version 1.0 (Szpiech et al. 2008), excluding loci with missing data greater than 0.2 for at least one population.

We tested the hypothesis that the genetic diversity of the traditional varieties decreases when the distance from the center of domestication increases. For this, a Spearman's correlation was performed using heterozygosity and distance from the centroid of the TMVB traditional varieties to the rest of the cultivated populations. Breeding line Blanco Tlaxcala and the cultivar from Spain were not included in this analysis.

### DETECTING GENE FLOW AND INTROGRESSION

Three approaches were used to assess gene flow and introgression: TreeMix (Pickrell and Pritchard 2012) to predict gene flow scenarios, Patterson's *D* statistic or the ABBA‐BABA test (Green et al. 2010; Durand et al. 2011) to evaluate the gene flow for the predicted scenarios and sympatric populations, and lastly, *f*_d_ statistic (Martin et al. 2015) to identify introgressed regions along the genome. These analyses were performed with the complete dataset (237 samples) and a sample subset (183 samples) to test if the different samples sizes affected the gene flow results. We used the individual bam and VCF files as input to run the ABBA‐BABA with ANGSD (Korneliussen et al. 2014) and Dsuite (Malinsky et al. 2021), respectively. For those combinations that were statistically significant for Patterson's *D*, we used the tool Dinvestigate from Dsuite to calculate *f*_d_ in sliding windows using 25 SNVs per window and a step size of 10 SNVs. Finally, *f*_d_ and the log10 of the *P*‐value for the *iHS* statistic (see SIGNATURES OF SELECTION WITHIN INTROGRESSED REGIONS section) were plotted along the 11 chromosomes.

The ABBA‐BABA approach is based on a resolved phylogeny among four taxa (((H1, H2), H3), H4) and determines if the pattern of derived alleles is consistent with the phylogeny (Green et al. 2010; Durand et al. 2011). To compute this test, we used bam files from each individual, and ran the analysis with the multipop ABBA‐BABA module from the package ANGSD (Korneliussen et al. 2014). Two of the three gene flow scenarios obtained with TreeMix (see *Results*) were evaluated with the ABBA‐BABA test: (1) gene flow from the wild population from Chiapas (Wild‐SUR‐CH) into the branch of all cultivars (Cult‐ancestral) and (2) gene flow from the Chiapas wild population (Wild‐SUR‐CH) into the Feral population (Table S2). Scenarios of sympatric gene flow from wild populations into traditional varieties and from *P. dumosus* cultivars into Chiapas wild runner bean populations (Wild‐SUR‐CH) were also evaluated. For all tested scenarios, wild *P. vulgaris* was used as an outgroup (H4), and the statistical significance (*P* < .05) was established after applying a Bonferroni correction to the block jackknife *P*‐value.

Because the ABBA‐BABA test assumes H3 (donor) diverged before the split between H1 and H2 (receptors), scenarios where a cultivar was a donor and a wild population was a receptor were excluded. Therefore, the third gene flow scenario suggested by TreeMix (introgression from the branch that clusters Cult‐TMVB and Cult‐SMOCC [Cult‐TMVB&SMOCC] into Wild‐TMVB‐CDMX, Fig. S5) was performed using Wild‐TMVB‐CDMX as H3 and Cult‐TMVB&SMOCC as H2 and Cult‐TMVB&MOCC as H2. In this case, the excess of shared alleles shows evidence as gene flow but not the direction of it.

### SIGNATURES OF SELECTION WITHIN INTROGRESSED REGIONS

To look for evidence of adaptive introgression, candidate regions under selection were identified in the three traditional varieties in which gene flow from the wild relatives was detected (see *Results*; Cult‐TMVB, Cult‐SUR‐CH, and Cult‐SMOCC). The candidate regions were then compared with the windows with the highest *f*_d_ values (5% top) per chromosome.

In the tested cultivated populations, recent selective sweeps were identified using the integrated haplotype homozygosity score (*iHS*; Gautier and Naves 2011; Gautier et al. 2017), which relays on the Extended Haplotype Homozygosity (*EHH*; Sabeti et al. 2002).

The most recent version of the rehh package was implemented because it is adapted for unphased data (Klassmann and Gautier 2022). The *P. vulgaris* alleles were set as the ancestral states. The analysis was performed on the three cultivated populations and to the 11 chromosomes independently. The minimum allele frequency (MAF) was 0.05 within each population, 25‐Kb windows and 12.5 Kb step size were fixed, the *iHS* threshold was fixed at 1.5, and *P* < 0.05. Genes found in candidate regions were annotated using MapMan4 (Schwacke et al. 2019).

### INFERRING THE DEMOGRAPHIC HISTORY

To find evidence of demographic processes that have affected *P. coccineus* populations, the Site Frequency Spectrum (SFS) of each population was constructed using the PLINK allele count function. Because the different SNV categories may be under different evolutionary processes, they provide complementary information. Therefore, we constructed the SFSs according to SNV in CDS, nongenic, and intronic regions. The expected SFS was derived by using the Watterson's estimator ${\hat{\theta }}_{W}\;=\;\frac{s}{\sum_{i\;=\;1}^{n-1}\;\frac{1}{i}}$. To identify signs of demographic bottlenecks, long Runs of Homozygosity (ROH) per individual were estimated with PLINK using a 500 Kb min window size.

Based on the SFSs, the ROHs, and the introgression events found with TreeMix and the ABBA‐BABA test, demographic scenarios were constructed and tested using fastsimcoal2 that uses coalescent simulations to model demographic scenarios from the SFS (Excoffier et al. 2013). Only nongenic regions were included to make the demographic inferences. For the Cult‐TMVB, Cult‐SMOCC, and Cult‐SUR‐CH, three scenarios were modeled. The models differ in the times when introgression from the wild relative occurred: recent (3000 generations ago to present), ancient (from 6000 to 3000 generations ago), and constant (6000 generations ago to present for Cult‐SMOCC and Cult‐SUR‐CH, and from divergence time to present for Cult‐TMVB). Additionally, bidirectional introgression was included for the TMVB populations during the first 2000 generations after divergence, which we considered as an early domestication phase.

In the demographic models of TMVB populations, the cultivated clade diverged from the wild one at TDOM (domestication onset) generations ago. For Cult‐SMOCC and Cult‐SUR‐CH models, we were interested in gene flow from sympatric wild relatives, even though such sympatric taxa are unlikely to be the closest wild populations. We thus expect that the time of divergence (TDIV) in these models will be earlier than the timing of domestication.

After an initial bottleneck in the cultivated population (NAC, ancestral population size), a demographic expansion occurred (NCC, current cultivated population size), at TEXP (time expansion) generations ago. The wild population size (NWILD) was assumed to be constant through time (Fig. S1). The migration rate from wild to domesticated populations was also modeled, being equal to MIGWC = NMWC/NWILD, where NMWC is the number of wild migrants and NWILD is the wild population size (Fig. S1). The domestication bottleneck was modeled only for the Cult‐TMVB population. For Cult‐SMOCC and Cult‐SUR‐CH, the modeled bottlenecks correspond to the traditional varieties spreading and it was assumed that they occurred after the initial domestication bottleneck. We did not find evidence of gene flow from wild relatives into Cult‐OV or Cult‐TMVB‐Spain (see *Results*); therefore, we only modeled the ancestral population size (NANC), the time when a bottleneck started (TBOT), population size during the bottleneck (NBOT), demographic expansion time (TEXP), and the current population size (NCUR; Fig. S1).

We ran 100,000 simulations with 100 independent replicates for each model. For the best‐fit model for those scenarios with gene flow, the likelihoods of the best runs were compared to estimate the AIC weight. Then we performed 50 bootstraps for the best‐fit model to obtain the mean and 95% confidence intervals for each parameter.

## Results

### SAMPLING AND SNV CALLING

After mapping, SNV calling, and filtering, 237 individuals of *P. coccineus* (89 wild, 131 cultivated, and 17 ferals), 20 of *P. vulgaris*, and 35 of *P. dumosus* were kept. The mean missing data were 1.22%, and the mean depth per site was 31.04×. The SNV dataset contained 79,286 SNVs, of which 11,019 variants were found in nongenic regions (13.90%), 35,429 within introns (44.68%), and 32,838 within CDS (41.42%). Regarding the variants within CDS, 13,738 (41.84%) were predicted as synonymous mutations, 18,392 (56.01%) as nonsynonymous, 541 (1.65%) as frameshift, and 248 as nonsense (0.75%).

To reduce the difference in sample size among the populations, a second filtering was applied based on relatedness and missingness. This dataset consisted of 183 individuals of *P. coccineus* and 71,861 SNVs, with a mean missing data 1.21% and mean depth per site of ∼32×.

### DEFINED POPULATIONS

**Figure 1:**
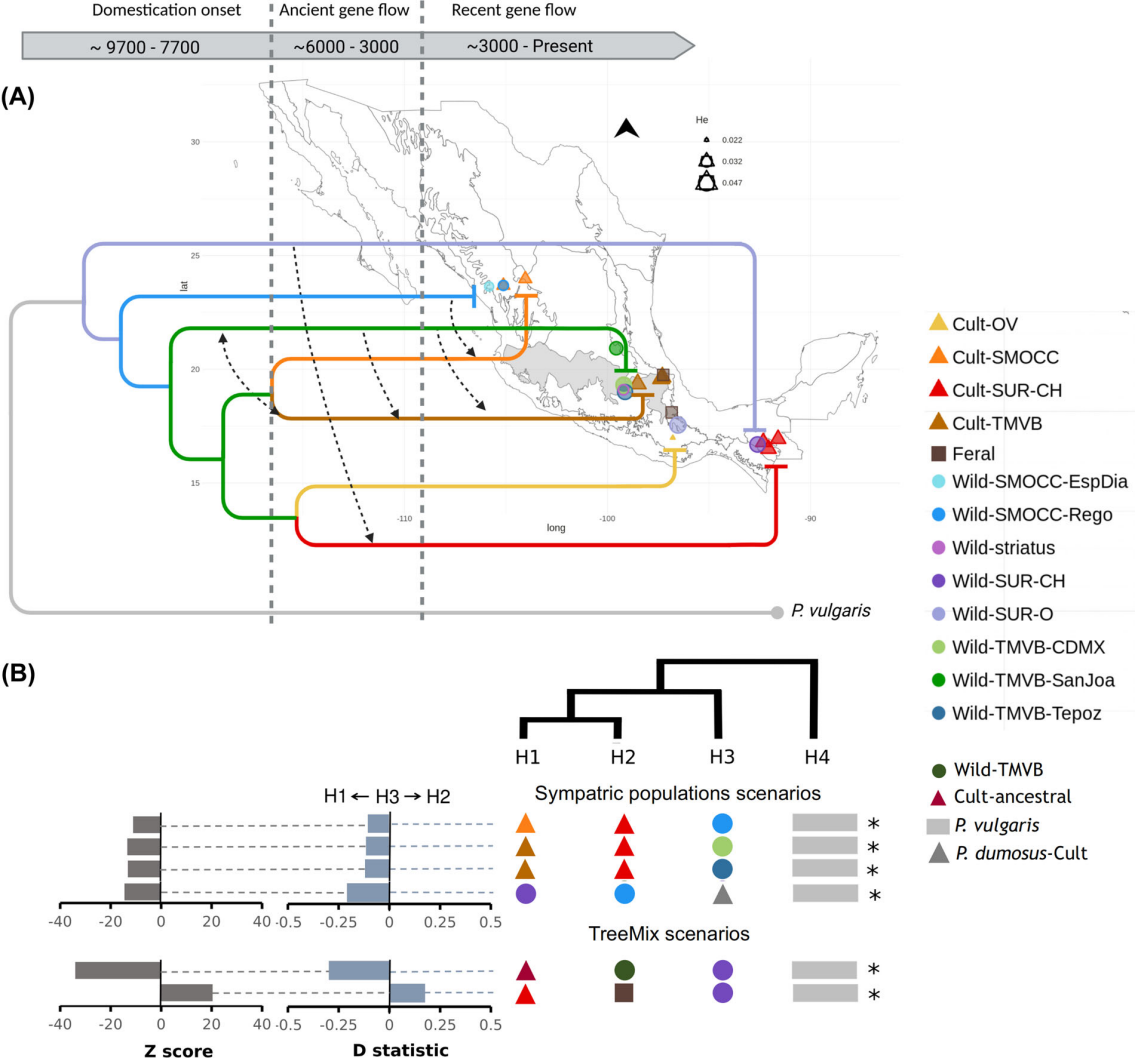
Population history of wild populations and traditional varieties of scarlet runner bean. (A) Distribution map of genotyped populations in Mexico. Circles indicate wild populations and triangles show traditional varieties. Boundaries represent 21 ecoregions as defined by CONABIO (2008). The tree shows the phylogenetic relationship between the populations, and arrows indicate the direction of gene flow over time. (B) Gene flow scenarios tested with the ABBA‐BABA test. The tree shows the phylogenetic relations assumed for the gene flow scenarios. Asterisks indicate statistical significance after Bonferroni correction (*P* < 0.05).

The 237 samples from these 24 geographic locations were grouped into 15 populations (Figs. 1A, S2) based on the phylogenetic tree constructed with FastTree and the PCA results (Figs. S1, S2B). The tree topology was similar to the one constructed by Guerra‐García et al. (2017). Cultivars formed a monophyletic clade, and wild populations from the TMVB were the closest to the domesticated group. Eight genetic groups were identified by Admixture using the 237 datasets, four of which corresponding to wild samples and the other half to traditional varieties (Fig. S3). Genetic clustering in our subsample of 183 individuals was very similar, with two of the cultivated clusters grouped together and some additional mixed ancestry identified in wild TMVB individuals from San Joaquín (Fig. S3). The 15 populations comprised four ecoregions of Mexico (as defined in CONABIO 2008). Eight of these populations were made up of by wild individuals: two from Sierra Madre del Sur and Chiapas Highlands (Oaxaca Wild‐SUR‐O; and Chiapas Wild‐SUR‐CH); three from Trans‐Mexican Volcanic Belt (Mexico City, Wild‐TMVB‐CDMX; San Joaquín, Wild‐TMVB‐SanJoa; Tepoztlán, Wild‐TMVB‐Tepoz); one identified as subsp. *striatus* (Wild‐striatus); and two from the Sierra Madre Occidental (Regocijo, Wild‐SMOCC‐Rego; Espinazo del Diablo, Wild‐SMOCC‐EspDia). The other six populations corresponded to cultivars: from Sierra Madre del Sur (Cult‐SUR‐CH); Oaxaca Valley (Cult‐OV); Trans‐Mexican Volcanic Belt (Cult‐TMVB); Sierra Madre Occidental (Cult‐SMOCC); the Spain cultivar, which was grouped within traditional varieties from TMVB in the ancestry analysis (Fig. S2; Cult‐TMVB‐Spain); and the breeding line Blanco‐Tlaxcala, with ancestry from the SMOCC cultivars (Fig. S2; Cult‐SMOCC‐BlaTla). All individuals identified as ferals were assigned to one group (Feral). The first word of the population name corresponds to the type of samples, followed by the genetic group assigned by Admixture, and the last letters indicate the population (see Table S1).

### WILD‐CROP INTROGRESSION AND SELECTION WITHIN INTROGRESSED REGIONS

Three gene flow events were proposed by TreeMix using the two datasets (Fig. S5): (1) from an ancestral cultivar lineage (Cult‐TMVB&SMOCC) to the TMVB wild population; (2) an ancient gene flow event from Wild‐SUR‐CH to an old clade that included all cultivars (Cult‐ancestral); and (3) from Wild‐SUR‐CH to ferals. The three scenarios were tested using the ABBA‐BABA method and were statistically supported (Fig. 1B; Tables S2 and S3), noting that in the case of the first scenario the direction of the gene flow could not be established with the ABBA‐BABA test.

We also looked for introgression among sympatric populations, and found evidence of gene flow from wild populations into cultivars: from Wild‐SMOCC‐Rego to Cult‐SMOCC; from Wild‐TMVB‐CDMX and Wild‐TMVB‐Tepoz into Cult‐TMVB; and from Wild‐SUR‐CH to Cult‐SUR‐CH (Fig. 1B). Sympatric populations of *P. dumosus* and *P. coccineus* occur in the Southern region of Mexico. We tested for introgression between these two species, and the ABBA‐BABA test supported introgression from *P. dumosus* to Wild‐SUR‐CH. Finally, the test showed evidence of frequent gene flow among traditional varieties, mainly from the Cult‐TMVB into the other traditional varieties (Tables S2–S4).

Introgressed regions along the genome from sympatric wild populations into traditional varieties of the TMVB, SMOCC, and SUR biogeographic regions were identified using the *f*_d_ statistic. The genomic windows with the highest 5% *f*_d_ were considered as introgressed. In the case of Cult‐SMOCC, 21 windows containing 122 genes were found, 28 windows and 176 genes in Cult‐SUR‐CH, and 73 windows comprising 386 genes in Cult‐TMVB (42 windows introgressed from Wild‐TMVB‐CDMX, and 31 windows from Wild‐TMVB‐Tepoz; Fig. S6). Search for selective sweeps was performed in the same three cultivated populations in which evidence of gene flow from the wild sympatric populations was found (Cult‐TMVB, Cult‐SMOCC, and Cult‐SUR). The number of selective sweeps and genes within those regions identified was as follows: 19 windows containing 19 genes in Cult‐TMVB; 20 windows and 18 genes within them in Cult‐SMOCC; and 21 windows with 19 in the case of Cult‐SUR (Table S5). Only a few of the candidate genes were located in introgressed regions: one in Cult‐SUR, four in Cult‐TMVB (two when the donor was Wild‐TMVB‐CDMX, and two from Wild‐TMVB‐Tepoz), and no genes that met both conditions were found in the Cult‐SMOCC (Fig. S7; Table S5).

Twenty‐eight out of the 56 candidate genes were annotated with MapMan4 (Table S5). The only gene in Cult‐SUR identified as a candidate for selection and introgression (Phvul.001G037500) was annotated as a solute transporter (NIPA). The four genes that met these two conditions in Cult‐TMVB were Phvul.008G287100 (nucleoporin), Phvul.009G184800 (formin actin filament elongation factor activities), Phvul.009G185000 (serine‐type peptidase activities, S16‐class protease), and Phvul.010G040300 (transferase transferring phosphorus‐containing group; Table S5).

### DEMOGRAPHIC HISTORIES OF TRADITIONAL VARIETIES

We constructed an SFS for each population and SNV category. Patterns varied among populations, suggesting that they have gone through different evolutionary processes (Fig. S8). Most of the wild populations presented a slight excess of low‐frequency alleles and subspecies *striatus* was the only wild population that showed a lack of low‐frequency variants (Fig. S8). Three traditional varieties and ferals also presented an excess of low‐frequency alleles (Cult‐SMOCC, Cult‐SUR, and Cult‐TMVB), whereas Cult‐TMVB‐Spain showed a deficit. Nonsynonymous mutations were the most abundant variants at low frequency in all populations (Fig. S8).

Higher ROH was found in cultivated populations compared to wild ones (Fig. S9). The European cultivar (Cult‐TMVB‐Spain) had the longest ROH, followed by a traditional variety from Oaxaca Valley (Cult‐OV). This suggests that cultivated populations have gone through demographic bottlenecks, but the excess of low‐frequency variants observed in several traditional varieties shows evidence of demographic expansions. Based on these results, an initial bottleneck followed by a demographic expansion was modeled for the cultivated populations using fastsimcoal2. The gene flow found from wild to cultivars from SMOCC, TMVB, and SUR‐CH were integrated into the demographic models, testing introgression at different times (ancient, recent, and constant; Figs. 1A, S1).

**Figure 2:**
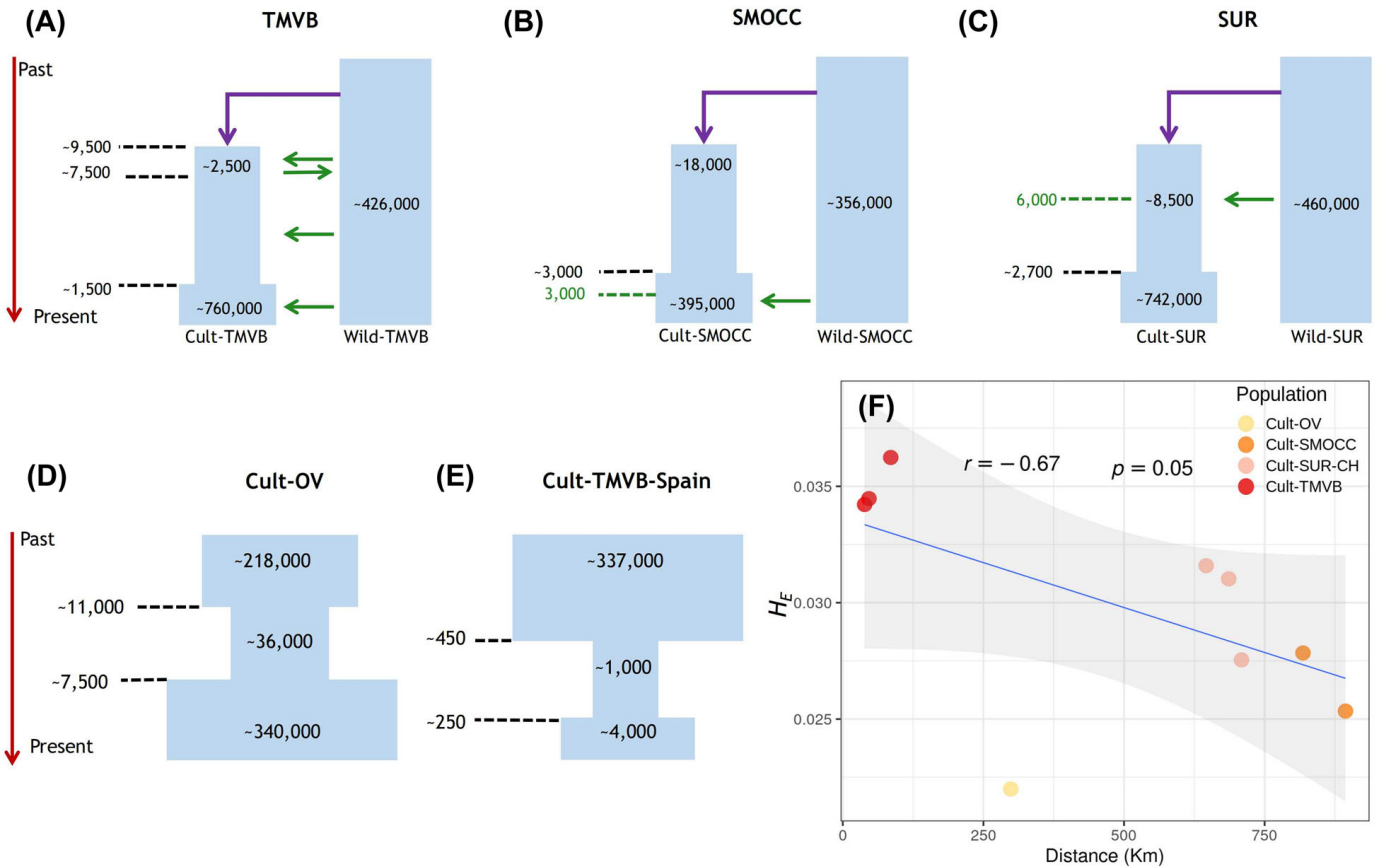
Best demographic scenarios and their parameters estimated with fastsimcoal2. (A) The best scenario for TMBV populations involves constant introgression; (B) recent introgression in the SMOCC populations; and (C) ancestral introgression in the case of populations from SUR‐CH. For all populations, the direction of the gene flow was from wild to cultivated beans. Ancestral bidirectional gene flow was included for the first 2000 generations after the beginning of domestication only for TMVB, where domestication took place. Demographic models for Cult‐OV (D) and Cult‐TMVB‐Spain (E), which have gone through bottlenecks in the absence of gene flow from wild populations. (F) Correlation between the genetic diversity (*H*_E_) and the distance from the centroid of the Cult‐TMVB locations to traditional variety locations.

The best‐fit scenario for Cult‐TMVB included constant introgression from the wild relatives to traditional varieties of this region, a severe bottleneck (NAC = ∼2500) associated with domestication time around 9700 generations ago, followed by a relatively recent expansion (TEXP = ∼1500), and a current population size of ∼759,000 (Figs. 1, 2; Table S6).

The best‐fit scenario for Cult‐SMOCC populations suggested a recent introgression (from 3000 generations ago to present), a less severe bottleneck associated with cultivar spreading (NAC = ∼18,000), and a current population size of ∼395,000. For the SUR‐CH, the best model predicted an ancient introgression (from 6000 to 3000 generations ago), a bottleneck that led to an ancestral population size of ∼8500, and a current population of ∼742,000 (Figs. 1, 2; Table S6). The Cult‐SUR‐CH population had the highest migration rate (MIGWC = 1.93 × 10^–5^) of the three populations (Table S6).

In the case of Cult‐OV and Cult‐TMVB‐Spain, only the severity and timing of the bottleneck were modeled. The bottleneck in Cult‐OV was not as severe as in all the other populations (NBOT = ∼36,000), and its current population size was estimated to be ∼340,000 (Fig. 2D; Table S6). In contrast, the bottleneck that Cult‐TMVB‐Spain went through was the most severe (NBOT = ∼1000) and the current population size remains relatively low (NCC = ∼4000; Fig. 2E; Table S6).

### MEASURING GENETIC DIVERSITY

Patterns of genetic diversity were similar between the full data and the subset of 183 individuals (Fig. S10; Table S7). Wild populations in general exhibited the highest diversity. The greatest amount of genetic diversity was found in both Wild‐SUR populations (Oaxaca and Chiapas) and Wild‐TMVB‐CDMX (Fig. S10). Nevertheless, the Cult‐TMVB and Feral populations also showed high levels of variation. Cultivars from Oaxaca, Spain, and the breeding line Blanco Tlaxcala had the lowest genetic diversity. All cultivars presented a higher inbreeding coefficient (*F*_IS_) than wild populations (Fig. S10; Table S7).

The highest values of differentiation were found between the cultivars and the wild relatives. The highest *F*_ST_ was estimated between Cult‐TMVB‐Spain and Wild‐SMOCC‐EspDia. The cultivars presented low differentiation among them, and the ferals showed relatively low *F*_ST_ with the wild and domesticated populations (Fig. S11). Similar levels of differentiation and the same patterns across populations were observed using the subsampled data (Fig. S11).

A large proportion of private alleles was observed (Fig. S12). Private alleles were classified as follows: private to wild populations, to cultivars, to ferals, and to each population. The last class of private alleles was the most abundant. Wild populations had a greater proportion of private alleles, with Wild‐SMOCC‐EspDia and Wild‐SUR‐O being the most notable. In both cases, 54% of their segregating sites were private to those populations. The lowest proportions were found in Cult‐TMVB‐Spain and Cult‐SMOCC‐BlaTla (9% in both). In CDS regions, the proportion of private alleles was 64% in the Wild‐SMOCC‐EspDia population and 54% in Wild‐SUR‐O.

Regarding the nonsynonymous/synonymous ratio, it was lower in the shared variants compared to the private ones, and it was particularly high in the cultivars (Table S8). The cultivars with the lowest nonsynonymous/synonymous ratio were Cult‐TMVB‐Spain and the breeding line Blanco Tlaxcala (Cult‐SMOCC‐BlaTla).

Rarefaction analyses showed greater allelic richness and private allelic richness in wild populations (Fig. S13), particularly in Wild‐TMVB‐CDMX, Wild‐TMVB‐Tepoz, and Wild‐SUR‐O, but it was also elevated in Feral and Cult‐TMVB populations. On the contrary, Cult‐TMVB‐Spain showed the lowest allelic richness.

A negative correlation was found between heterozygosity in traditional varieties and the distance to the centroid of the area where TMVB traditional varieties were collected (Spearman correlation [*r*_S_] = −0.67, *P* < 0.05 for the 237 datasets, Fig. 2F; and *r_S_* = −0.68, *P* = 0.05 for the 183 samples subset, Fig. S14).

## Discussion

### DEMOGRAPHIC HISTORIES OF SCARLET RUNNER BEAN POPULATIONS

Genome‐wide comparisons between wild and cultivars have been studied in several crops (e.g., He et al. 2011; Huang et al. 2012; Hufford et al. 2012; Cavanagh et al. 2013; Li et al. 2013; Zhou et al. 2015; Bellucci et al. 2014; Schmutz et al. 2014; Vlasova et al. 2016; Rendón‐Anaya et al. 2017; Wang et al. 2018). These have shown that the severity of bottlenecks varies substantially among species, within species, and even among gene pools.

Wild common bean has the widest geographic distribution (from northern Mexico to Argentina) compared to the other domesticated bean species (Ariani et al. 2017). Numerous population genetics and demographic analysis have been performed in *P. vulgaris*, showing that the patterns of population size and variation differ substantially depending on the gene pools and geographic region (Chacón et al. 2007; Kwak and Gepts 2009; Bitocchi et al. 2013; Mamidi et al. 2013; Schmutz et al. 2014; Ariani et al. 2017). These studies have shown a strong bottleneck associated with domestication, and a severe pre‐domestication bottleneck in the Andean gene pool. Furthermore, climate factors that have shaped the distribution of wild common bean have been identified (Ariani et al. 2017). In spite of that, more information and studies focusing on the history of the wild and domesticated populations of other domesticated bean species are needed to properly use their genetic resources.

The demographic inferences of the scarlet runner bean performed in this work showed that each population presents a unique history, with different severity and timing of bottleneck and expansion. Moreover, we detected that introgression from wild relatives into cultivars is frequent, and it has occurred at different rates and times across populations.

Despite the relatively high genetic diversity found in Cult‐TMVB, the best demographic model suggests a strong bottleneck related to domestication and constant introgression from wild populations that might have contributed to increasing levels of genetic variation. Furthermore, the Cult‐TMVB population has increased its size, allowing the accumulation of new mutations, which are most likely private and are at low frequency, making it difficult to detect them, because they appeared recently. The estimated domestication time is reasonable (9700 generations ago), even though it is higher than that for common bean, in which it started ∼8000 year ago (Gepts 1998; Kwak et al. 2009), but the oldest archaeological records date back 2285 BP (Tehuacán Caves) and 2098 BP (Oaxaca Valley; Kaplan and Lynch 1999), and linguistic analyses suggest an origin of at least 3400 years (Brown et al. 2014). Nonetheless, at Guilá Naquitz Cave, also located in Oaxaca, a type of morphologically wild bean was present between 10,600 and 8500 BP in quantities that might suggest that people artificially increased their density by cultivating them (Flannery 1986).

Our results suggest that the introgression from wild relatives has only taken place during the last 3000 generations in the sympatric SMOCC populations. Meanwhile, the introgression in the SUR‐CH seems to be older (6000–3000 generations ago; Figs. 1, 2) and at a higher migration rate (Table S6). The Cult‐SUR‐CH current population size is almost the same as Cult‐TMVB, indicating a conspicuous expansion (Fig. 2B; Table S6). Interestingly, the Cult‐OV population, where gene flow from Cult‐TMVB and Cult‐SMOCC was detected, had the least severe bottleneck but also the lowest genetic diversity among Mexican traditional varieties.

The estimated bottleneck for Cult‐TMVB‐Spain agrees with our expectation and occurred after the foundation of the Viceroyalty of New Spain in 1525. During this time, an intense bidirectional exchange went on between Spain and what is now Mexico. The introduction of scarlet runner beans to Europe resulted in an intense bottleneck and, even though a population increase occurred, its population size is still low compared to Mexican traditional varieties. Because just one European cultivar was analyzed, however, no general pattern can yet be inferred about scarlet runner beans in Europe.

### FREQUENT AND ASYMMETRIC INTROGRESSION FROM WILD RELATIVES INTO TRADITIONAL VARIETIES

Introgression is frequent among *P. coccineus* populations, which may be facilitated by the sympatry of wild and domesticated populations. Our results suggest that gene flow from wild to traditional varieties is a frequent event, and just one gene flow scenario from cultivar (Cult‐TMVB and Cult‐SMOCC) into a wild population (Wild‐TMVB‐CDMX) was inferred using TreeMix (Fig. S5). With the ABBA‐BABA test, we found evidence of this last scenario, but the directions of the gene flow could not be assessed with this approach.

Contrasting with the results found in this study, asymmetric gene flow from crop to wild has been reported in common bean (Papa and Gepts 2003) and lima bean (Félix et al. 2014), resulting in the displacement and reduction of genetic diversity of the wild relatives. Papa et al. (2005) found a significantly higher differentiation between wild relatives and cultivars in allopatric populations compared to sympatric ones. Furthermore, differentiation was higher in genes related to domestication, suggesting that selection was preventing introgression from domesticated into wild forms at target loci. In other regions, introgression was larger due to the lack of selection against domesticated maladapted genes (Papa et al. 2005). The asymmetric gene flow in common bean was confirmed with genomic data (Rendón‐Anaya et al. 2017). Another process that might be playing a role in the asymmetric gene flow is the presence of alleles related to nuclear‐cytoplasmic conflict, causing cross incompatibility between wild and domesticated populations. This has been described in pea (*Pisum sativum*), another important legume crop (Bogdanova et al. 2015; Nováková et al. 2019).

Introgression from wild to traditional varieties could be a source of adaptive variation because crop dispersion implies adaptation to new environments, and because wild populations are presumably adapted to local conditions (Janzen et al. 2019). Adaptive introgression may explain the patterns of asymmetric gene flow observed in *P. coccineus* and has probably maintained a relatively high genetic diversity of traditional varieties. Nevertheless, the overlapping between *f*_d_ and *iHS* along the *P. coccineus* genome (Fig. S6) is low and very few of the candidate genes under selection in the traditional varieties were also present in the introgressed genomic windows (Fig. S7; Table S5). This could be due to the data resolution reached with the GBS approach that was implemented to genotype the samples included in this work. Because GBS consists of a sampling of the genome, the SNV density yielded might not be optimum for selection tests (Tiffin and Ross‐Ibarra 2017). Furthermore, none of the candidate genes found in this work were reported in the previous study (Guerra‐Garcia et al. 2017). The hypothesis of adaptive introgression in this domesticated species could be further explored with a higher density genotyping method, performing a targeted sequencing approach, and/or functional genomics.

Because a recurrent goal in breeding programs is the introgression of adaptive traits from wild relatives into cultivated (Warschefsky et al. 2014), these already introgressed traditional varieties from three different wild pools become a powerful resource for valuable agronomic traits dissection. Crop‐wild introgressed populations contain a mixture of wild and crop alleles that can be valued as an in situ germplasm resource in comparison with nonintrogressed populations (Ellstrand 2018).

### PURIFYING SELECTION ACTING IN WILD AND DOMESTICATED POPULATIONS

Most wild populations and traditional varieties showed an excess of low‐frequency variants (Fig. S8). Furthermore, in all populations, the proportion of nonsynonymous at low frequency is higher than the SNVs within nongenic regions and synonymous mutations. This is the expected pattern under purifying selection, which keeps deleterious alleles from increasing in the population (Nielsen and Slatkin 2013). The nonsynonymous/synonymous ratio of the segregating sites tends to be >1 in all wild and cultivated populations, except in Cult‐TMVB‐Spain. This might be caused by the private segregating sites, which were common in the populations and presented an even higher nonsynonymous/synonymous ratio (Table S8). These private variants were probably recently originated in both wild and cultivated populations, noting that “recent” for both population types refers to different time intervals. This might suggest the presence of genetic load both in wild populations and traditional varieties, mainly integrated by recent private variants.

The cultivars that showed the lowest nonsynonymous/synonymous ratio were Cult‐TMVB‐Spain (0.957), followed by the breeding line Blanco Tlaxcala (Cult‐SMOCC‐BlaTla, 1.037). Two different processes that followed the severe bottleneck in these populations (caused by the introduction to Europe or the sampling to create the breeding line) might explain their relatively low ratio: the bottleneck removed low‐frequency variants, which have the highest nonsynonymous/synonymous ratio, decreasing the ratio, and/or the strong artificial selection possibly resulted in the expression and posterior purge of some deleterious alleles. González et al. (2014) reported inbreeding depression in European scarlet runner bean cultivars, which affected germination, survival rates, yield, and seed weight. This may indicate that although a genetic purge might have occurred, deleterious variants associated with complex or quantitative traits were maintained. When inbreeding depression is caused by a small number of recessive alleles with major deleterious effects on fitness, rapid response to selection is expected. However, deleterious variants with small effects are less easily purged and can be maintained in the population (Byers and Waller 1999; Charlesworth and Willis 2009; Samayoa et al. 2021).

### GENETIC DIVERSITY

The greatest estimates of genetic diversity were found in wild populations. Nevertheless, there are wild populations with lower genetic diversity than traditional varieties, such as both Wild‐SMOCC and Wild‐striatus. The expectation that high diversity will be maintained close to the center of domestication and decrease with increasing geographic distance is observed in our data (Fig. 2F). This supports the hypothesis proposed by Guerra‐García et al. (2017) that domestication took place in the TMVB, which was the most diverse traditional variety. Conversely, populations that have gone through subsequent bottlenecks have had a shorter time to accumulate new variation, such as the cultivar from Spain (Cult‐TMVB‐Spain) and Blanco Tlaxcala breeding line (Cult‐SMOCC‐BlaTla). Additionally, probably these two populations have gone through stronger artificial selection.

The proportion of private alleles is lower in traditional varieties compared to their wild relatives, but still significant. In Cult‐SUR and Cult‐TMVB, private variation represents 33% of their segregating sites. The demographic expansions of the traditional varieties that occurred after the domestication bottlenecks have probably favored the emergence of new variants.

Populations with the highest genetic diversity did not have the greatest private variation. This was the case of Wild‐TMVB‐CDMX. In contrast, the proportion of segregating sites that were private to Wild‐SMOCC‐EspDia and Wild‐SUR‐O was remarkably high (46% for both groups). The presence of private alleles might be at least partially explained by population histories. An ancient population, with high population sizes, would show a high proportion of private alleles (Nielsen and Slatkin 2013). This could be the case of the wild and highly diverse populations from Southern Mexico. But in Wild‐SMOCC‐EspDia, the private alleles are at low frequencies, suggesting that they emerged recently in terms of the origin of the wild populations, although likely pre‐domestication. The demography of the wild populations of scarlet runner bean remains elusive and a future demographic study of the wild relatives would complement and allow a deeper understanding of the genetic variation in *P. coccineus* as a species.

Furthermore, it has been suggested that domestication bottlenecks might be less severe for perennials than for annual plants because perennial species frequently have a cross‐pollination mating system and overlapping generations (Gaut et al. 2015). Although scarlet runner bean is commonly cultivated as an annual crop, it is a perennial open‐pollinated species, and occasionally its subterraneous structures are kept for the next agricultural cycle (Delgado‐Salinas 1988). In this work, we have provided evidence of demographic expansions and introgression from the wild relatives into cultivated *P. coccineus*. Both demography and gene flow have played key roles in promoting and maintaining genetic diversity in scarlet runner bean at its center of domestication.

## AUTHOR CONTRIBUTIONS

AGG and DP planned and designed the research and interpreted the data. AGG and IRB conducted fieldwork, analyzed the data, and wrote the manuscript. JRI and RP contributed to the data interpretation and discussion.

## DATA ARCHIVING

The data that support the findings of this study are openly available in the Open Science Framework (OSF) at https://osf.io/h7sa5/ and the scripts are at https://github.com/AzaleaGuerra/Wild‐to‐crop_introgression_Pcocci.

## CONFLICT OF INTEREST

The authors declare no conflict of interest.

## Supporting information


**Table S1**. *Phaseolus coccineus* sampling material used. “Population” column refers to the 15 defined populations that were the clustering factor in the analysis. All samples corresponded to *P. coccineus* subsp. c*occineus*, except a wild population from Tres Marías, Morelos. The last column shows the number of individuals that were kept after variant filtering.
**Fig. S1**. Demographic models that were tested using fastsimcoal2. For TMVB, SMOCC and SUR populations recent, constant and ancestral gene flow were tested. The likelihood for each of these scenarios are shown in the right column. For Cult‐OV and the populations from Spain the severity and time of the bottleneck was estimated. NWILD= *Ne* wilds; NCC= Current *Ne* cultivars; NAC= Ancestral *Ne* cultivars; NBOT= *Ne* during the bottleneck; TBOT= bottleneck time (generations); TEXP= time of demographic expansion; TDOM= domestication time; TDIV= divergence time.
**Fig. S2**. a) Phylogenetic relationship among individuals of *P. coccineus* from Mexico. The 15 defined populations are indicated and colors in the ancestry plot do not correspond to the colors of the 15 populations. b) PCA plot for the first two principal components including all samples.
**Fig. S3**. Ancestry plots of the wild and traditional varieties of *P. coccineus*. The ancestry analysis was performed using the complete data set and the data subset (183 samples). The 15 defined populations are indicated in the extreme right of the ancestry plots.
**Fig. S4**. PCA plots for the first two components of a) cultivated and b) wild populations.
**Fig. S5**. Gene flow scenarios inferred by TreeMix. a) Analysis performed using complete data set, and b) with the data subset consisting of 183 samples.
**Table S2**. Gene flow models tested for cultivated, feral and wild populations of *P. coccineus* using the ABBA‐BABA approach.
**Table S3**. Gene flow models tested with a sample subset for cultivated, feral and wild populations of *P. coccineus* with the ABBA‐BABA test. Simple size is indicated with (n) in the corresponding columns.
**Table S4**. Gene flow scenarios tested with Dsuite.
**Fig. S6**. Introgressed regions and under positive selection along the 11 chromosomes from the reference genome of *P. vulgaris*. The left axis shows the log_10_(p‐values) for the selection statistic *iHS* (blue solid line) and the right axis shows the values for the fd statistic (dotted red line) computed in sliding windows of 25,10 SNVs per window and step. a) Introgression from Wild‐SUR‐CH into Cult‐SUR‐CH, b) Introgression from Wild‐SMOCC‐Rego into Cult‐SMOCC, c) Introgression from Wild‐TMVB‐CDMX into Cult‐TMVB, and d) Introgression from Wild‐TMVB‐Tepoz into Cult‐TMVB. The gray line shows the statistical significance threshold for iHS ( log_10_(0.05) = 1.3).
**Fig. S7**. Venn diagram showing the number of genes found in the introgressed regions (blue circles) and candidate genes under selection (yellow circles) in the three traditional varieties in which gene flow from the wild sympatric populations was detected.
**Table S5**. Genes found in the candidate regions of the three evaluated traditional varieties. The *Donor population* column shows if the gene was also presented in the introgressed region identified.
**Fig. S8**. Site Frequency Spectrum (SFS) calculated for each population. Colors indicate the SNV category. Points show the expected distribution.
**Fig. S9**. Total length of ROH (Kb) estimated using a 500 Kb min window size. Colors indicate the type of *P. coccineus* sample.
**Table S6**. Demographic parameters estimated using fastsimcoal2 (95% CI). NWILD= *Ne* wilds; NCC= Current *Ne* cultivars; NAC= Ancestral *Ne* cultivars; NBOT= Ne during the bottleneck; TBOT= bottleneck time (generations); TEXP= time of demographic expansion; TDOM= domestication time; TDIV= divergence time; REXP= expansion rate; MIGWC= migration rate from wild to cult (NMWC/NWILD); MIGCW= migration rate from cult to wild (NMCW/NAC); NMWC= migrants from wild to cult; NMCW= migrants from cult to wild.
**Fig S10**. Heatmaps showing the genetic diversity in terms of *H* and inbreeding coefficient estimated for the 15 defined populations using the 237 samples.
**Table S7**. Genetic diversity levels in the *P. coccineus* populations. Expected (*H_E_*) and observed (*H_O_*) heterozygosity, and inbreeding coefficient (*F_IS_*). The confidence intervals were obtained performing 100 bootstraps. Asterisk shows the estimated values using the data subset (183 samples).
**Fig. S11**. Differentiation index among the 15 populations of *P. coccineus*. a) was estimated using the complete data set, and b) with the data subset (183 samples).
**Fig. S12**. a) Proportion of private and shares alleles for each population. b) Proportion of private and shared alleles within CDS regions, separating into synonymous and nonsynonymous mutations. The numbers inside the columns indicate the proportion of each category. Only segregating sites within populations are included.
**Table S8**. Proportion of segregating sites (SS) and nonsynonymous/synonymous ratio of SS splitted into the shared and private alleles found within each *P. coccineus* population.
**Fig. S13**. A) Mean allelic richness per site and (B) mean private alleles per site estimated with AZDE for each population.
orrelation between the genetic diversity (*H_E_*) and the distance from the centroid of the Cult‐TMVB locations to traditional variety locations estimated using the data subset (
**Fig. S14**. Correlation between the genetic diversity (*H_E_*) and the distance from the centroid of the Cult‐TMVB locations to traditional variety locations estimated using the data subset (183 samples).
