# Supplementary material for: The genomic signature of wild‐to‐crop introgression during the domestication of scarlet runner bean (Phaseolus coccineus L.)
Source: Evol Lett. 2022 Jun 15;6(4):295–307. doi: 10.1002/evl3.285 (PMC9346085; doi:10.1002/evl3.285)
Supplement: Supplementary file 1 — Table S1. Phaseolus coccineus sampling material used. “Population” column refers to the 15 defined populations that were the clustering factor in the analysis. All samples corresponded to P. coccineus subsp. coccineus, except a wild population from Tres Marías, Morelos. The last column shows the number of individuals that were kept after variant filtering. Fig. S1. Demographic models that were tested using fastsimcoal2. For TMVB, SMOCC and SUR populations recent, constant and ancestral gene flow were tested. The likelihood for each of these scenarios are shown in the right column. For Cult‐OV and the populations from Spain the severity and time of the bottleneck was estimated. NWILD= Ne wilds; NCC= Current Ne cultivars; NAC= Ancestral Ne cultivars; NBOT= Ne during the bottleneck; TBOT= bottleneck time (generations); TEXP= time of demographic expansion; TDOM= domestication time; TDIV= divergence time. Fig. S2. a) Phylogenetic relationship among individuals of P. coccineus from Mexico. The 15 defined populations are indicated and colors in the ancestry plot do not correspond to the colors of the 15 populations. b) PCA plot for the first two principal components including all samples. Fig. S3. Ancestry plots of the wild and traditional varieties of P. coccineus. The ancestry analysis was performed using the complete data set and the data subset (183 samples). The 15 defined populations are indicated in the extreme right of the ancestry plots. Fig. S4. PCA plots for the first two components of a) cultivated and b) wild populations. Fig. S5. Gene flow scenarios inferred by TreeMix. a) Analysis performed using complete data set, and b) with the data subset consisting of 183 samples. Table S2. Gene flow models tested for cultivated, feral and wild populations of P. coccineus using the ABBA‐BABA approach. Table S3. Gene flow models tested with a sample subset for cultivated, feral and wild populations of P. coccineus with the ABBA‐BABA test. Simple size is indicated with (n [file EVL3-6-295-s001.docx]

Table S1. *Phaseolus coccineus* sampling material used. “Population” column refers to the 15 defined populations that were the clustering factor in the analysis. All samples corresponded to *P. coccineus* subsp. c*occineus*, except a wild population from Tres Marías, Morelos. The last column shows the number of individuals that were kept after variant filtering.

| **State and location** | **Status** | **Population** | **Lat.** | **Long.** | **Altitude** | | ***n*** | ***n**** |
| --- | --- | --- | --- | --- | --- | --- | --- | --- |
| *Phaseolus coccineus* | | | | | |  |  |  |
| Oaxaca, Cuilapam | Cultivar | Cult-OV | 16.99 | -96.78 | 1577 | | 13 | 12 |
| Durango | Cultivar | Cult-SMOCC | NA | NA | NA | | 8 | 7 |
| Durango, Regocijo | Cultivar | Cult-SMOCC | 23.68 | -105.12 | 2566 | | 5 | 2 |
| Durango, Villa Unión | Cultivar | Cult-SMOCC | 23.97 | -104.04 | 1901 | | 12 | 11 |
| Blanco Tlaxcala | Breeding line | Cult-SMOCC-BlTl | NA | NA | NA | | 12 | 11 |
| Chiapas, González León | Cultivar | Cult-SUR-CH | 16.51 | -92.06 | 1579 | | 10 | 5 |
| Chiapas, Nahá | Cultivar | Cult-SUR-CH | 16.94 | -91.59 | 917 | | 12 | 9 |
| Chiapas, Oxchuc | Cultivar | Cult-SUR-CH | 16.80 | -92.32 | 2001 | | 12 | 6 |
| Veracruz, Frijol Colorado | Cultivar | Cult-TMVB | 19.59 | -97.35 | 2419 | | 11 | 4 |
| Veracruz, Orilla del Monte | Cultivar | Cult-TMVB | 19.66 | -97.29 | 2402 | | 10 | 7 |
| Puebla, Tlalancaneca | Cultivar | Cult-TMVB | 19.36 | -98.51 | 2372 | | 12 | 5 |
| Puebla | Cultivar | Cult-TMVB | NA | NA | NA | | 6 | 3 |
| Spain | Cultivar | Cult-TMVB-Spain | NA | NA | NA | | 8 | 8 |
| Veracruz, Altotonga | Feral | Feral | 19.75 | -97.25 | 1959 | | 9 | 6 |
| Oaxaca, Huautla | Feral | Feral | 18.10 | -96.83 | 1746 | | 8 | 8 |
| Durango, Espinazo del Diablo | Wild | Wild-SMOCC-EspDia | 23.64 | -105.82 | 1422 | | 11 | 11 |
| Durango, Regocijo | Wild | Wild-SMOCC-Rego | 23.68 | -105.12 | 2566 | | 12 | 12 |
| Morelos, Tres Marías | Wild subsp. *striatus* | Wild-striatus | 19.10 | -99.21 | 3024 | | 16 | 13 |
| Chiapas, San Cristóbal | Wild | Wild-SUR-CH | 16.70 | -92.60 | 2229 | | 7 | 6 |
| Oaxaca, Comaltepec | Wild | Wild-SUR-O | 17.55 | -96.53 | 2876 | | 4 | 4 |
| Mexico City, REPSA | Wild | Wild-TMVB-CDMX | 19.32 | -99.20 | 2328 | | 11 | 8 |
| Ciudad de México, Tlalpan | Wild | Wild-TMVB-CDMX | 19.29 | -99.19 | 2334 | | 8 | 7 |
| Querétaro, San Joaquín | Wild | Wild-TMVB-SanJoa | 20.93 | -99.56 | 2381 | | 6 | 6 |
| Morelos, Tepoztlán | Wild | Wild-TMVB-Tepoz | 19.00 | -99.13 | 1953 | | 13 | 12 |
| *Phaseolus vulgaris* | | | | | |  |  |  |
| Jalisco | Wild |  | NA | NA | NA | | 2 | NA |
| Mexico City, REPSA | Wild |  | 19.32 | -99.19 | 2328 | | 2 | NA |
| Morelos, Yautepec | Wild |  | 18.95 | -99.08 | 1386 | | 4 | NA |
| *Phaseolus dumosus* | | | | | |  |  |  |
| Veracruz, Altotonga | Cultivar |  | 19.75 | -97.25 | 1959 | | 5 | NA |
| Puebla, Cuetzalan | Cultivar |  | 19.99 | -97.54 | 1684 | | 12 | NA |
| Oaxaca, Huautla | Cultivar |  | 18.10 | -96.83 | 1746 | | 4 | NA |
| Chiapas, Chiquinivalvo | Cultivar |  | 16.71 | -92.86 | 1467 | | 6 | NA |
| Chiapas, Motozintla | Cultivar |  | 15.43 | -92.33 | 2671 | | 6 | NA |


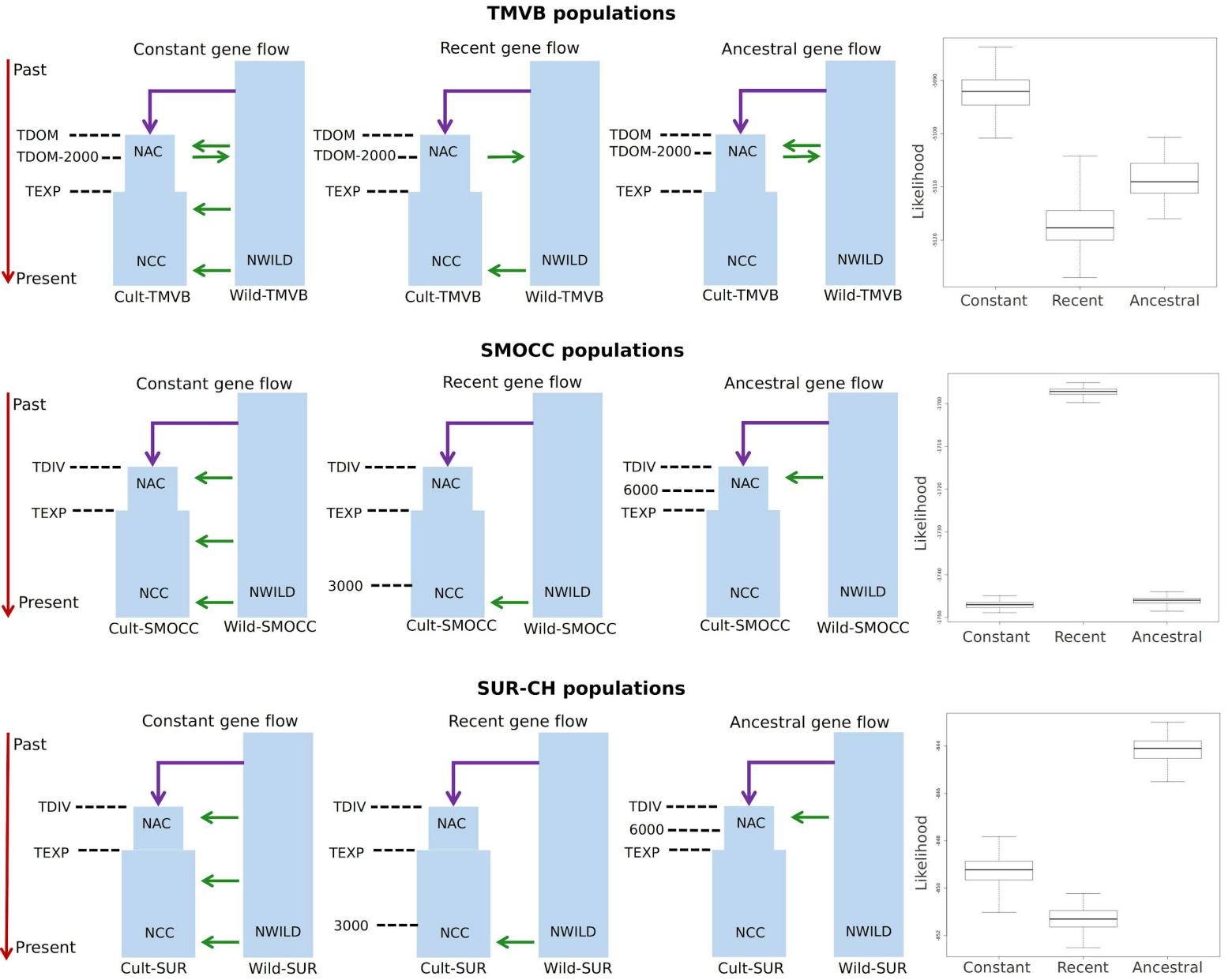


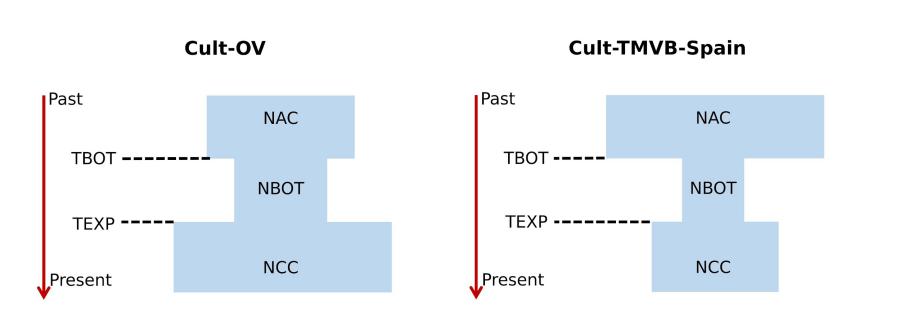


**Fig. S1.** Demographic models that were tested using fastsimcoal2. For TMVB, SMOCC and SUR populations recent, constant and ancestral gene flow were tested. The likelihood for each of these scenarios are shown in the right column. For Cult-OV and the populations from Spain the severity and time of the bottleneck was estimated. NWILD= *Ne* wilds; NCC= Current *Ne* cultivars; NAC= Ancestral *Ne* cultivars; NBOT= *Ne* during the bottleneck; TBOT= bottleneck time (generations); TEXP= time of demographic expansion; TDOM= domestication time; TDIV= divergence time.


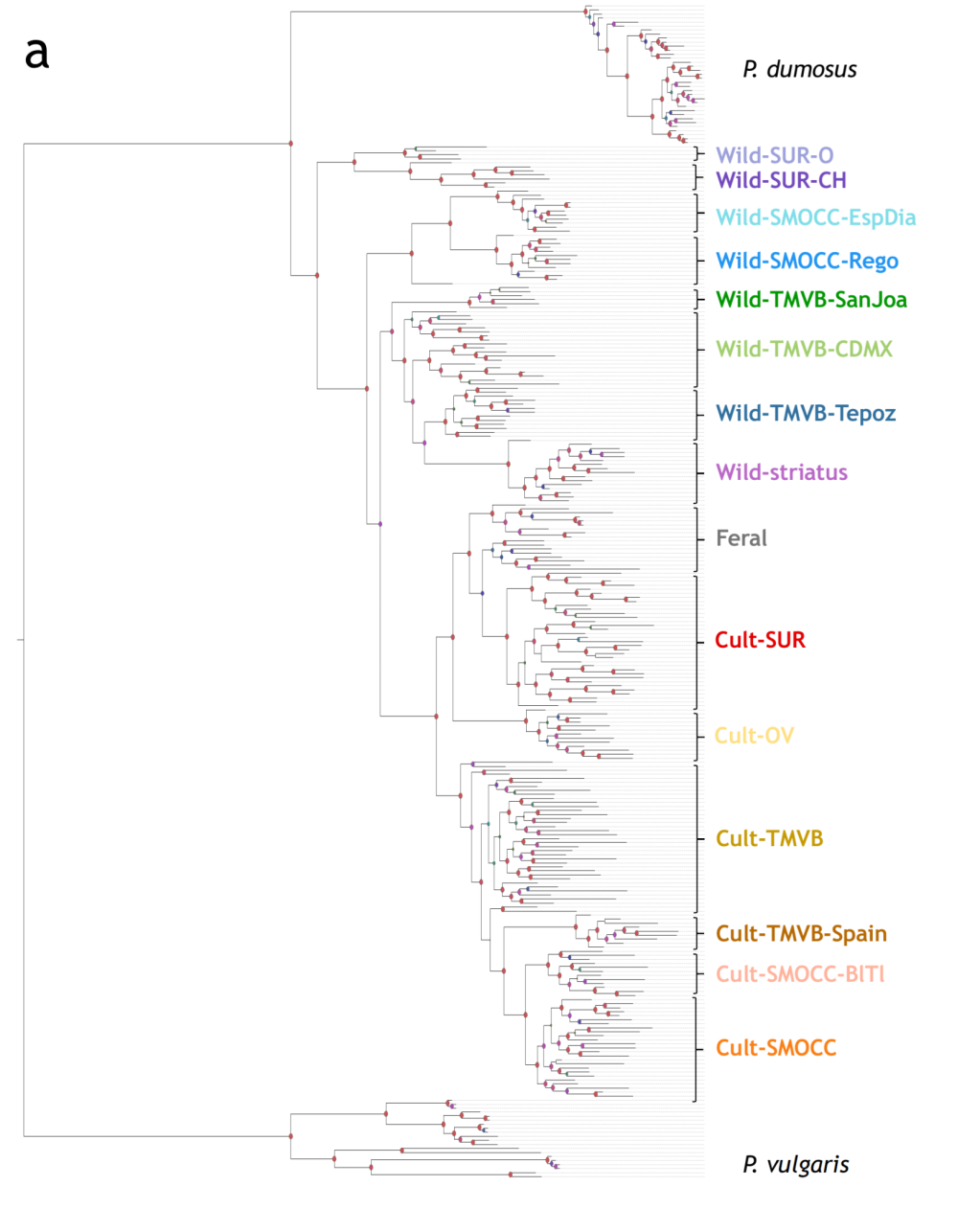


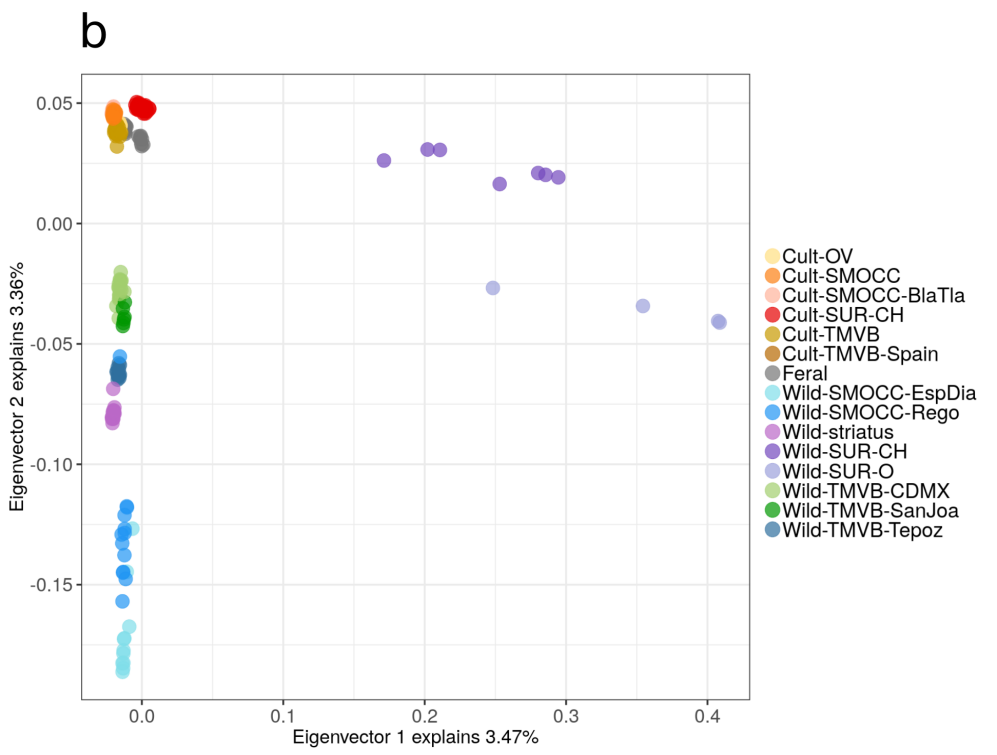


Fig. S2. a) Phylogenetic relationship among individuals of *P. coccineus* from Mexico. The 15 defined populations are indicated and colors in the ancestry plot do not correspond to the colors of the 15 populations. b) PCA plot for the first two principal components including all samples.


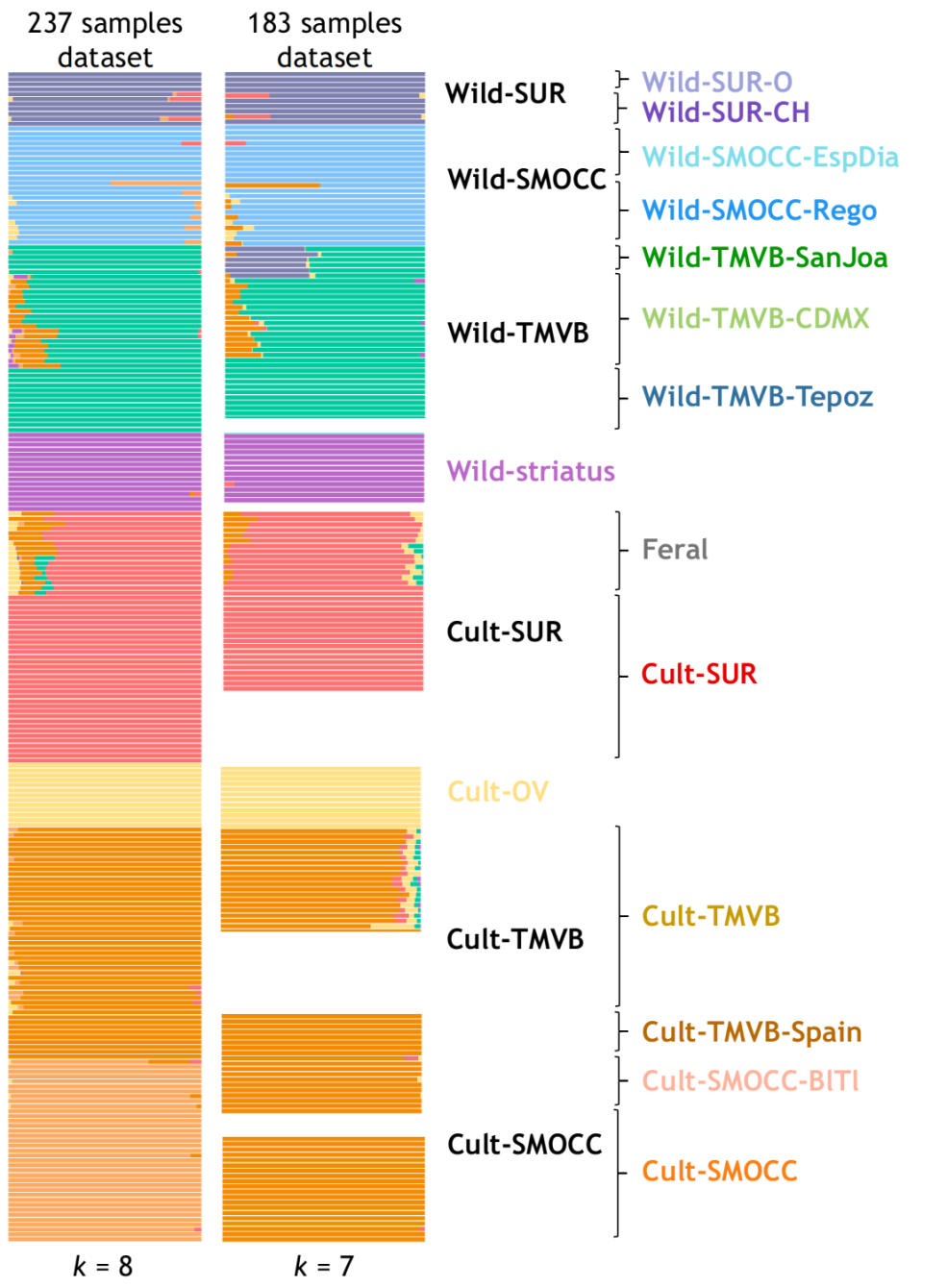


**Fig. S3.**  Ancestry plots of the wild and traditional varieties of *P. coccineus*. The ancestry analysis was performed using the complete data set and the data subset (183 samples). The 15 defined populations are indicated in the extreme right of the ancestry plots.


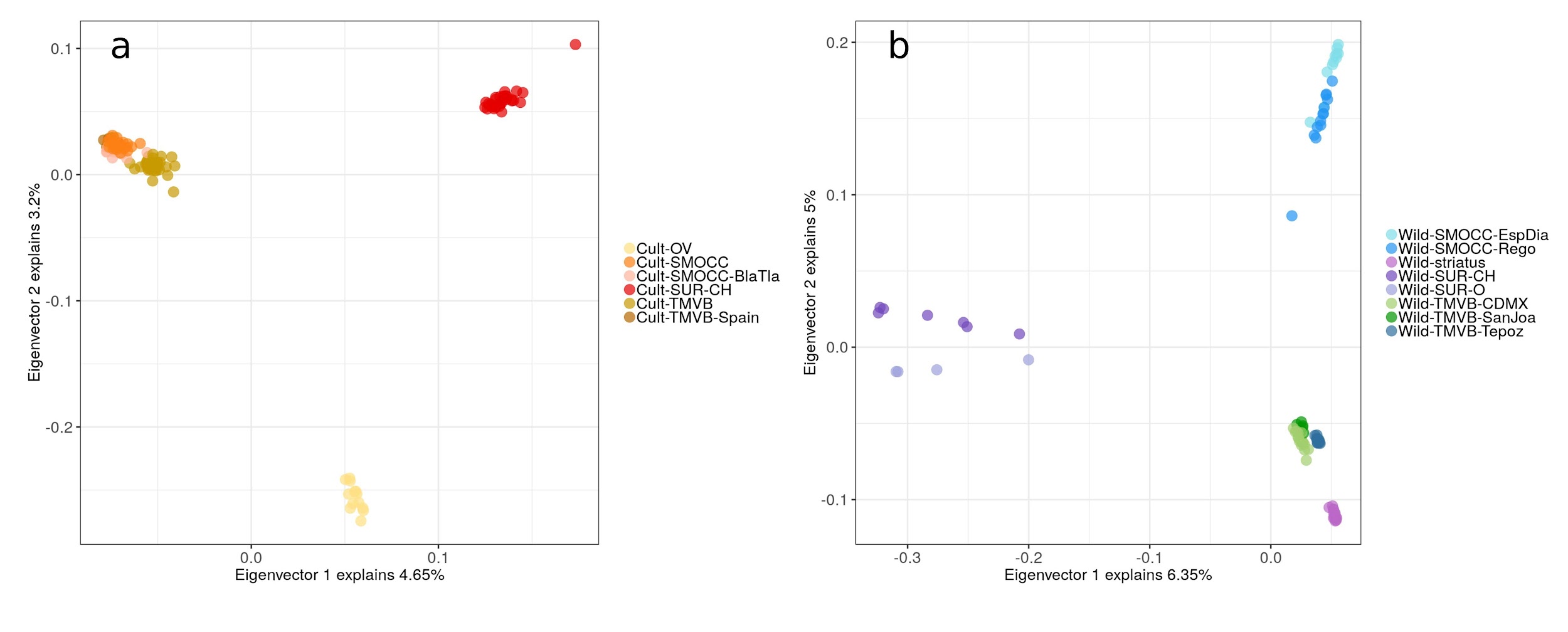

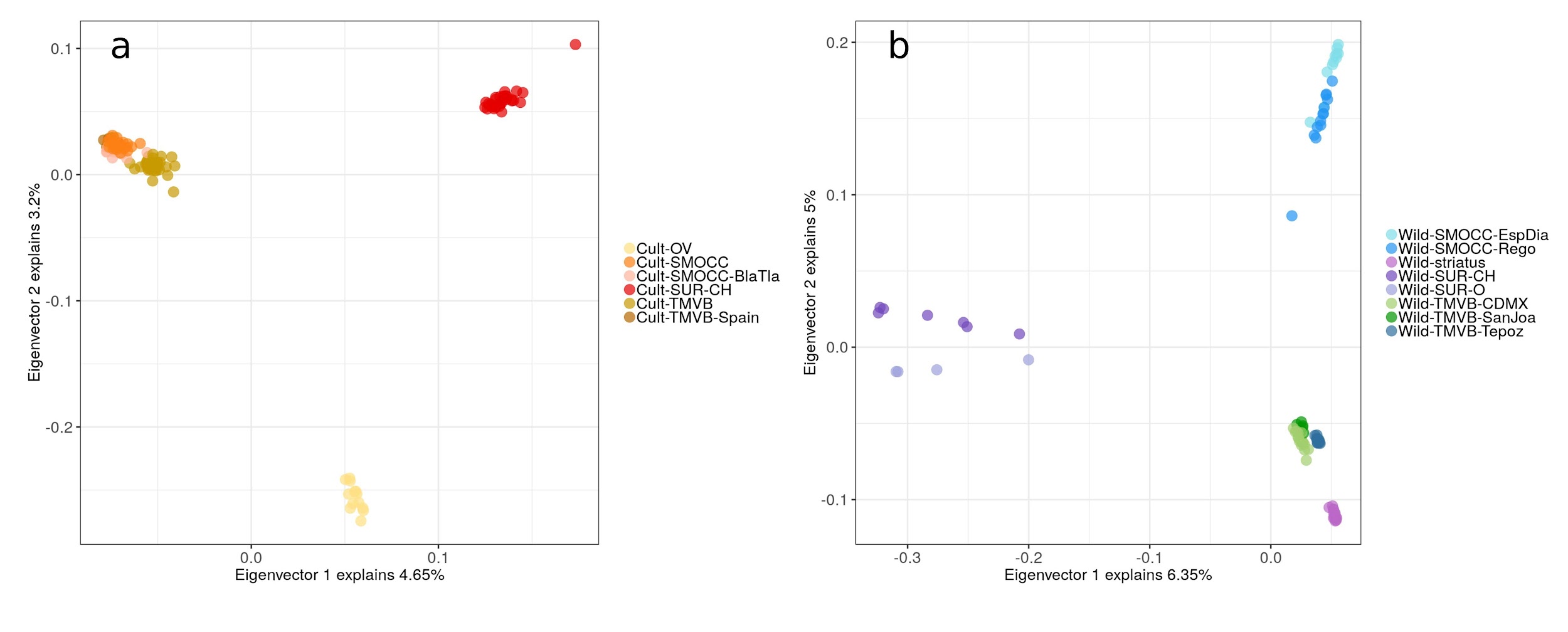


**Fig. S4.** PCA plots for the first two components of a) cultivated and b) wild populations.


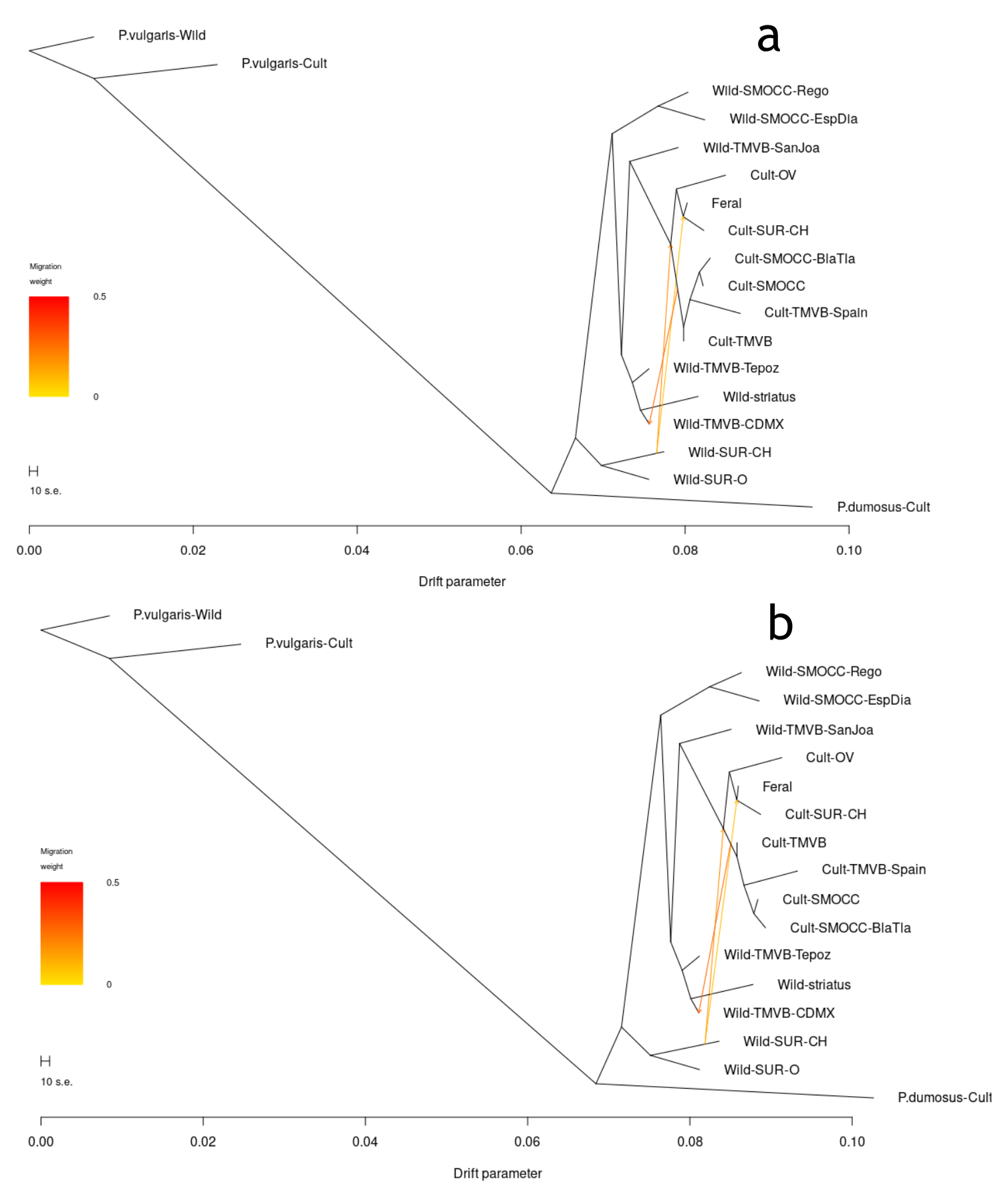


Fig. S5. Gene flow scenarios inferred by TreeMix. a) Analysis performed using complete data set, and b) with the data subset consisting of 183 samples.

Table S2. Gene flow models tested for cultivated, feral and wild populations of *P. coccineus* using the ABBA-BABA approach.

| **H1** | **H2** | **H3** | **H4** | **D** | **Z** | ***p value*** | **Bonferroni *p* value** | **Statistical significance** |
| --- | --- | --- | --- | --- | --- | --- | --- | --- |
|  |  |  |  |  |  |  |  |  |
| Cult-SUR-CH | Feral | Wild-SUR-CH | *P.vulgaris-Wild* | 0.176 | 20.397 | 0 | 0 | * |
| Feral | Cult-SUR-CH | Cult-TMVB | *P.vulgaris-Wild* | 0.009 | 1.053 | 0.2923 | 0.8771 | Not |
| Cult-SMOCC | Cult-SUR-CH | Wild-SMOCC-Rego | *P.vulgaris-Wild* | -0.105 | -10.908 | 0 | 0 | * |
| Cult-TMVB | Cult-SUR-CH | Wild-TMVB-CDMX | *P.vulgaris-Wild* | -0.113 | -13.239 | 0 | 0 | * |
| Cult-TMVB | Cult-SUR-CH | Wild-TMVB-Tepoz | *P.vulgaris-Wild* | -0.118 | -13.024 | 0 | 0 | * |
| Cult-SUR-CH | Cult-OV | Wild-SUR-CH | *P.vulgaris-Wild* | -0.148 | -14.643 | 0 | 0 | * |
| Cult-SUR-CH | Cult-OV | Cult-TMVB | *P.vulgaris-Wild* | 0.045 | 4.604 | 0 | 0 | * |
| Cult-SMOCC | Cult-TMVB-Spain | Cult-TMVB | *P.vulgaris-Wild* | -0.021 | -2.238 | 0.0252 | 0.0757 | Not |
| Cult-SUR-CH | Cult-OV | Cult-SMOCC | *P.vulgaris-Wild* | 0.036 | 3.348 | 0.0008 | 0.0024 | * |
| Wild-SUR-CH | Wild-SMOCC-Rego | *P. dumosus-*Cult | *P.vulgaris-Wild* | -0.208 | -14.592 | 0 | 0 | * |
| Cult-TMVB&SMOCC | Cult-SUR-CH | Wild-SUR-CH | *P.vulgaris-Wild* | 0.173 | 17.836 | 0 | 0 | * |
| Cult-ancestral | Wild-TMVB | Wild-SUR-CH | *P.vulgaris-Wild* | -0.298 | -34.042 | 0 | 0 | * |
| Cult-OV | Cult-TMVB&SMOCC | Wild-TMVB-CDMX | *P.vulgaris-Wild* | 0.049 | 6.159 | 0 | 0 | * |
| Cult-SUR-CH | Cult-TMVB&SMOCC | Wild-TMVB-CDMX | *P.vulgaris-Wild* | 0.108 | 12.652 | 0 | 0 | * |

Table S3. Gene flow models tested with a sample subset for cultivated, feral and wild populations of *P. coccineus* with the ABBA-BABA test. Simple size is indicated with (n) in the corresponding columns.

| **H1 (n)** | **H2 (n)** | **H3 (n)** | **H4 (n)** | **D** | **Z** | ***p* value** | **Bonferroni *p* value** |
| --- | --- | --- | --- | --- | --- | --- | --- |
| Cult-SUR-CH (20) | Feral (17) | Wild-SUR-CH (6) | *P.vulgaris*-Wild (8) | 0.1709 | 19.22 | 0.0000 | 0 |
| Feral (17) | Cult-SUR-CH (20) | Cult-TMVB (19) | *P.vulgaris*-Wild (8) | 0.0082 | 0.9054 | 0.3652 | 1.460828 |
| Cult-SMOCC (20) | Cult-SUR-CH (20) | Wild-SMOCC-Rego (12) | *P.vulgaris*-Wild (8) | -0.0990 | -9.9197 | 0.0000 | 0 |
| Cult-TMVB (19) | Cult-SUR-CH (20) | Wild-TMVB-CDMX (15) | *P.vulgaris*-Wild (8) | -0.1086 | -13.0010 | 0.0000 | 0 |
| Cult-TMVB (19) | Cult-SUR-CH (20) | Wild-TMVB-Tepoz (12) | *P.vulgaris*-Wild (8) | -0.1087 | -12.0862 | 0.0000 | 0 |
| Cult-SUR-CH (20) | Cult-OV (12) | Wild-SUR-CH (6) | *P.vulgaris*-Wild (8) | -0.1444 | -13.2711 | 0.0000 | 0 |
| Cult-SUR-CH (20) | Cult-OV (12) | Cult-TMVB (19) | *P.vulgaris*-Wild (8) | 0.0511 | 5.1446 | 0.0000 | 0 |
| Cult-SMOCC (20) | Cult-TMVB-Spain (8) | Cult-TMVB (19) | *P.vulgaris*-Wild (8) | -0.0382 | -3.8820 | 0.0001 | 0.0004 |
| Cult-SUR-CH (20) | Cult-OV (12) | Cult-SMOCC (20) | *P.vulgaris*-Wild (8) | 0.0390 | 3.6729 | 0.0002 | 0.0009 |
| Wild-SUR-CH (6) | Wild-SMOCC-Rego (12) | *P.dumosus*-Cult (35) | *P.vulgaris*-Wild (8) | -0.2137 | -15.0178 | 0.0000 | 0 |
| Cult-TMVB-SMOCC (57) | Cult-SUR-CH (20) | Wild-SUR-CH (6) | *P.vulgaris*-Wild (8) | 0.0812 | 9.81562 | 0.0000 | 0 |
| Cult-ancestral (94) | Wild-TMVB (33) | Wild-SUR-CH (6) | *P.vulgaris*-Wild (8) | -0.2970 | -32.5132 | 0.0000 | 0 |

Table S4. Gene flow scenarios tested with Dsuite.

| **H1** | **H2** | **H3** | **Dstatistic** | **f4-ratio** | **Z-score** | **p-value** | **Statistical significance** |
| --- | --- | --- | --- | --- | --- | --- | --- |
| Cult-SUR-CH | Feral | Wild-SUR-CH | 0.1508620 | 0.1165810 | 9.1570600 | 0.00000 | * |
| Feral | Cult-SUR-CH | Cult-TMVB | 0.0181207 | 0.0724019 | 1.3237400 | 0.18559 | Not |
| Cult-SUR-CH | Cult-SMOCC | Wild-SMOCC-Rego | 0.1252540 | 0.0784718 | 7.2040600 | 0.00000 | * |
| Cult-SUR-CH | Cult-TMVB | Wild-TMVB-CDMX | 0.1093390 | 0.4218880 | 8.3717100 | 0.00000 | * |
| Cult-SUR-CH | Cult-TMVB | Wild-TMVB-Tepoz | 0.1078350 | 0.1561450 | 6.9523600 | 0.00000 | * |
| Cult-OV | Cult-SUR-CH | Wild-SUR-CH | 0.1809490 | 0.1357840 | 10.5712000 | 0.00000 | * |
| Cult-SUR-CH | Cult-OV | Cult-TMVB | 0.0524022 | 0.2369210 | 2.9677000 | 0.00300 | * |
| Cult-SMOCC | Cult-TMVB-Spain | Cult-TMVB | 0.0008093 | 0.0000000 | 0.0439146 | 0.96497 | Not |
| Cult-SUR-CH | Cult-OV | Cult-SMOCC | 0.0319817 | 0.0700179 | 1.7422800 | 0.08146 | * |
| Wild-SMOCC-Rego | Wild-SUR-CH | *P. dumosus*-Cult | 0.2791520 | 0.0756597 | 8.6792200 | 0.00000 | * |
| Cult-TMVB-SMOCC | Cult-SUR-CH | Wild-SUR-CH | 0.1862780 | 0.1402280 | 11.6052000 | 0.00000 | * |


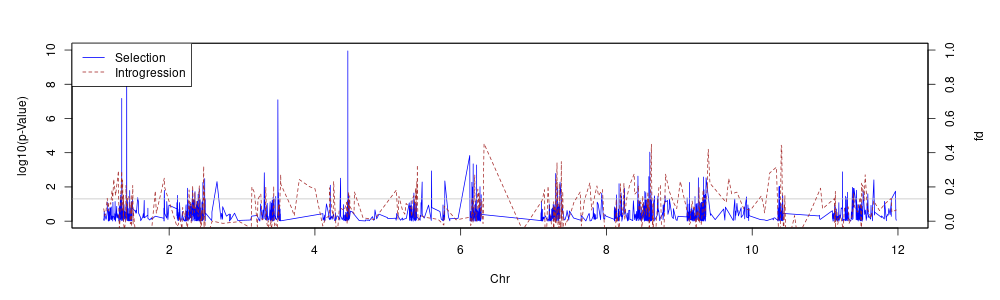


a


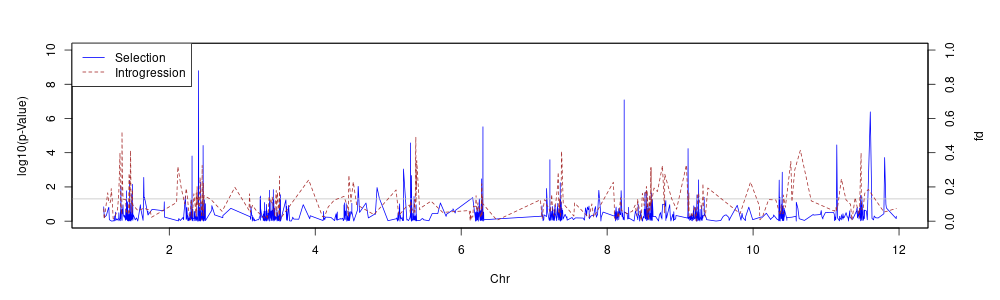


b

c
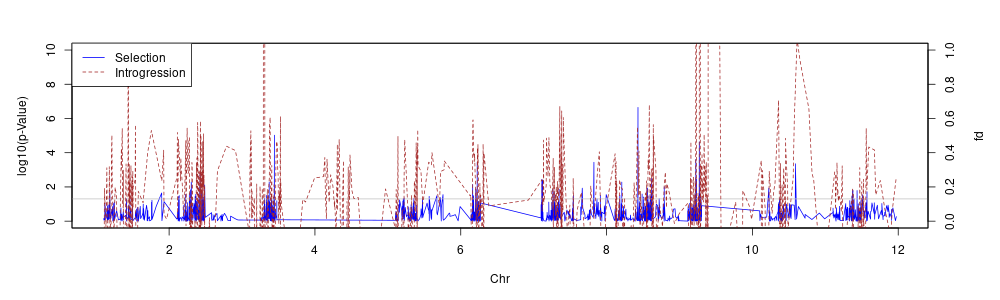


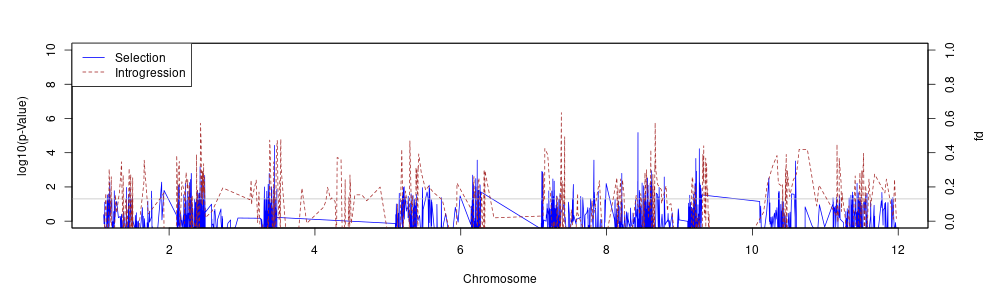


d

**Fig. S6.** Introgressed regions and under positive selection along the 11 chromosomes from the reference genome of *P. vulgaris*. The left axis shows the log_10_(p-values) for the selection statistic *iHS* (blue solid line) and the right axis shows the values for the fd statistic (dotted red line) computed in sliding windows of 25,10 SNVs per window and step. a) Introgression from Wild-SUR-CH into Cult-SUR-CH, b) Introgression from Wild-SMOCC-Rego into Cult-SMOCC, c) Introgression from Wild-TMVB-CDMX into Cult-TMVB, and d) Introgression from Wild-TMVB-Tepoz into Cult-TMVB. The gray line shows the statistical significance threshold for iHS ( log_10_(0.05) = 1.3).


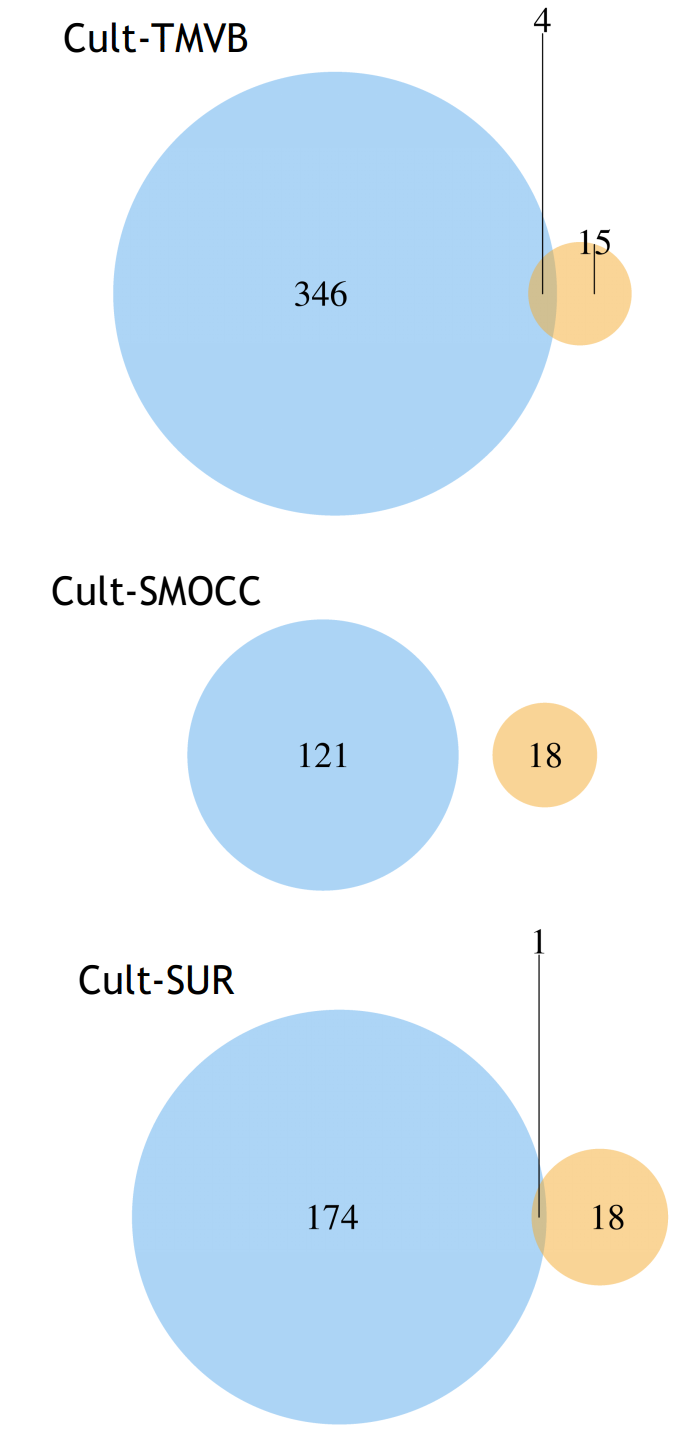


**Fig. S7**. Venn diagram showing the number of genes found in the introgressed regions (blue circles) and candidate genes under selection (yellow circles) in the three traditional varieties in which gene flow from the wild sympatric populations was detected.

**Table S5**. Genes found in the candidate regions of the three evaluated traditional varieties. The *Donor population* column shows if the gene was also presented in the introgressed region identified.

| **Population** | **Gene ID** | **Donor population** | **MapMan4 annotation** |
| --- | --- | --- | --- |
| Cult-SMOCC | Phvul.002G045700 |  |  |
|  | Phvul.002G046200 |  | Nucleotide sugar biosynthesis, UDP-D-glucose 4-epimerase |
|  | Phvul.003G201800 |  |  |
|  | Phvul.005G023400 |  | Polyamine metabolism, polyamine degradation, cytosolic polyamine oxidase (PAO1) |
|  | Phvul.005G033000 |  | Meiotic crossover, class II interference-insensitive crossover pathway, MUS81-dependent pathway, MUS81-EME1 Holliday junction cleavage heterodimer, component MUS81 |
|  | Phvul.005G033600 |  |  |
|  | Phvul.006G178500 |  |  |
|  | Phvul.006G201600 |  |  |
|  | Phvul.006G202200 |  | Plant reproduction, flowering, floral meristem identity control, regulatory protein (SVP/AGL24) |
|  | Phvul.007G098800 |  | Exocytic trafficking, Exocyst complex, component SEC8 |
|  | Phvul.008G029200 |  | Cell wall organisation, cellulose-hemicellulose network assembly, regulatory protein (COB) |
|  | Phvul.008G183500 |  |  |
|  | Phvul.009G149700 |  |  |
|  | Phvul.010G094600 |  |  |
|  | Phvul.010G119400 |  | Multi-pathway trafficking regulation, vesicle tethering, HOPS/CORVET tethering complexes, CORVET-specific component VPS8 |
|  | Phvul.010G119700 |  | Multi-process regulation, circadian clock system, time-of-day-dependent expressed repressor (PRR) |
|  | Phvul.010G142900 |  |  |
|  | Phvul.011G032100 |  |  |
| Cult-SUR-CH | Phvul.001G037500 | Wild-SUR-CH | Carrier-mediated transport, DMT superfamily, solute transporter (NIPA) |
|  | Phvul.001G042300 |  | RNA polymerase II-dependent transcription, transcription co-activation, TAF module shared with SAGA/TFIId complexes, component TAF5 |
|  | Phvul.002G053700 |  | Cytoskeleton organisation, microtubule Tubulin heterodimer formation, gamma-Tubulin Ring Complex (gamma-TuRC), component GCP6 |
|  | Phvul.004G017900 |  | Mitochondrial RNA splicing, splicing factor (SLO3) |
|  | Phvul.004G157500 |  |  |
|  | Phvul.006G055500 |  |  |
|  | Phvul.006G055800 |  | Carrier-mediated transport, ZIP family, metal cation transporter (ZIP) |
|  | Phvul.008G024500 |  |  |
|  | Phvul.008G024800 |  |  |
|  | Phvul.008G196300 |  |  |
|  | Phvul.008G230200 |  |  |
|  | Phvul.008G245800 |  |  |
|  | Phvul.008G265700 |  | DNA polymerase (POP) |
|  | Phvul.009G086400 |  | Protein modification, PPM/PP2C Mn/Mg-dependent phosphatase families, clade I phosphatase |
|  | Phvul.009G218900 |  | Protein homeostasis, proteolysis, serine-type peptidase activities, S8-class protease (subtilisin) families, protease (SBT2) |
|  | Phvul.009G231400 |  | Chromatin organisation, histone chaperone (NAP) |
|  | Phvul.010G134100 |  | Large ribosomal subunit (LSU) processome, pre-60S ribosomal subunit nuclear export, ATP-dependent export factor (MDN1) |
|  | Phvul.010G134400 |  |  |
|  | Phvul.011G027550 |  | Cellular respiration, NADH dehydrogenase complex, assembly factor (NDUFAF7) |
| Cult-TMVB | Phvul.002G037800 |  |  |
|  | Phvul.002G038300 |  |  |
|  | Phvul.002G045700 |  |  |
|  | Phvul.002G046200 |  | Nucleotide sugar biosynthesis, UDP-D-glucose 4-epimerase |
|  | Phvul.006G094900 |  | Cellular respiration, glycolysis, cytosolic glycolysis, phosphoglycerate mutase |
|  | Phvul.007G225600 |  |  |
|  | Phvul.008G021400 |  |  |
|  | Phvul.008G022000 |  |  |
|  | Phvul.008G025300 |  | Multi-process regulation, phosphatidylinositol and inositol phosphate system, phosphatidylinositol 4-kinase (PI4K-gamma) |
|  | Phvul.008G048800 |  |  |
|  | Phvul.008G049100 |  |  |
|  | Phvul.008G049200 |  |  |
|  | Phvul.008G054800 |  | Transcription factor (C3H-ZF) |
|  | Phvul.008G270200 |  |  |
|  | Phvul.008G274700 |  | Cytoskeleton organisation, Kinesin microtubule-based motor protein activities (Kinesin-12) |
|  | Phvul.008G287100 | Wild-TMVB-CDMX | Protein translocation, nuclear pore complex (NPC), outer ring, scaffold nucleoporin (NUP96/MOS3) |
|  | Phvul.009G184800 | Wild-TMVB-Tepoz | Cytoskeleton organisation, formin actin filament elongation factor activities, group-II formin |
|  | Phvul.009G185000 | Wild-TMVB-Tepoz | Protein homeostasis, proteolysis, serine-type peptidase activities, S16-class protease (LON) |
|  | Phvul.010G040300 | Wild-TMVB-CDMX | Enzyme classification, transferase transferring phosphorus-containing group |


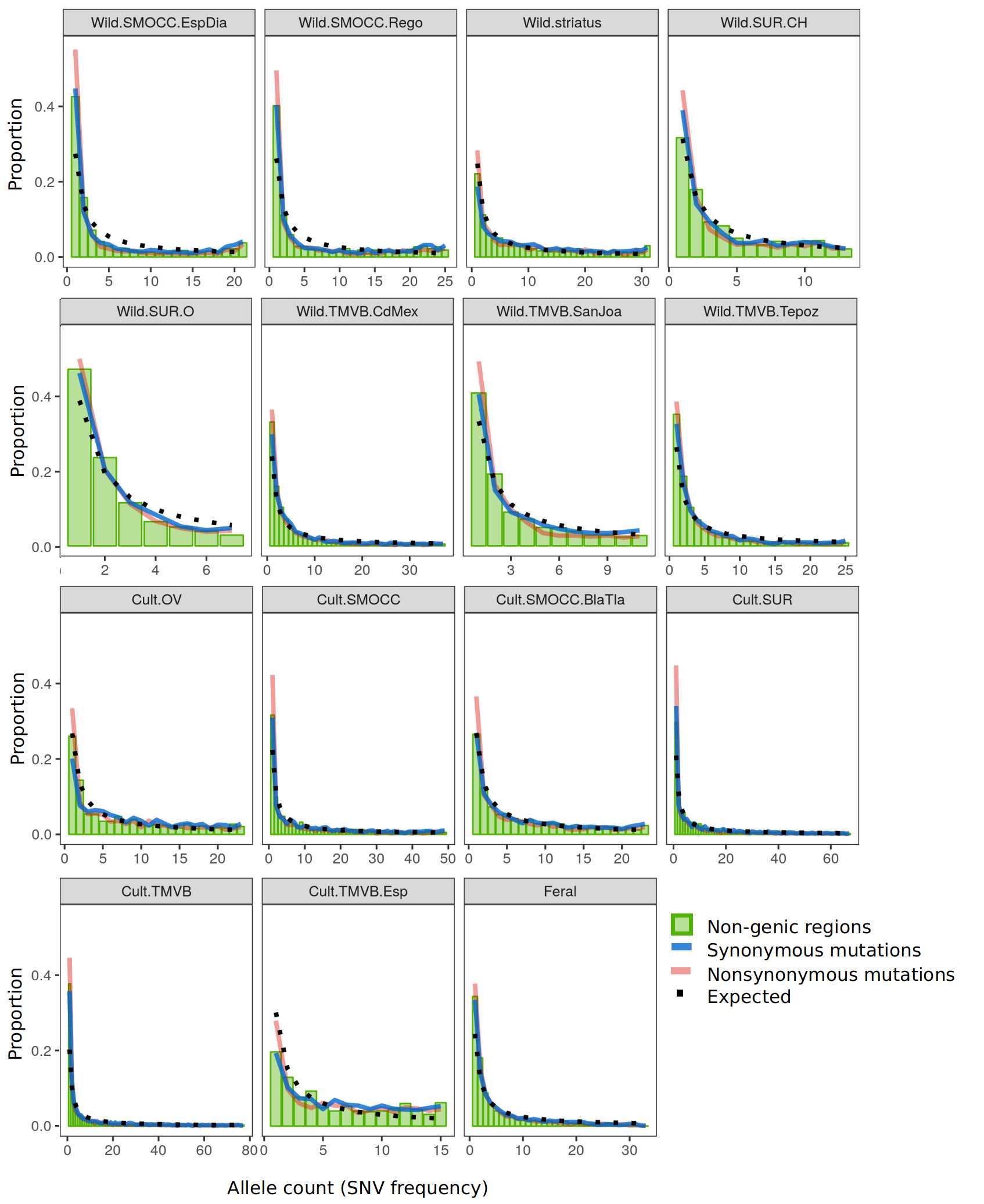


**Fig. S8**. Site Frequency Spectrum (SFS) calculated for each population. Colors indicate the SNV category. Points show the expected distribution.


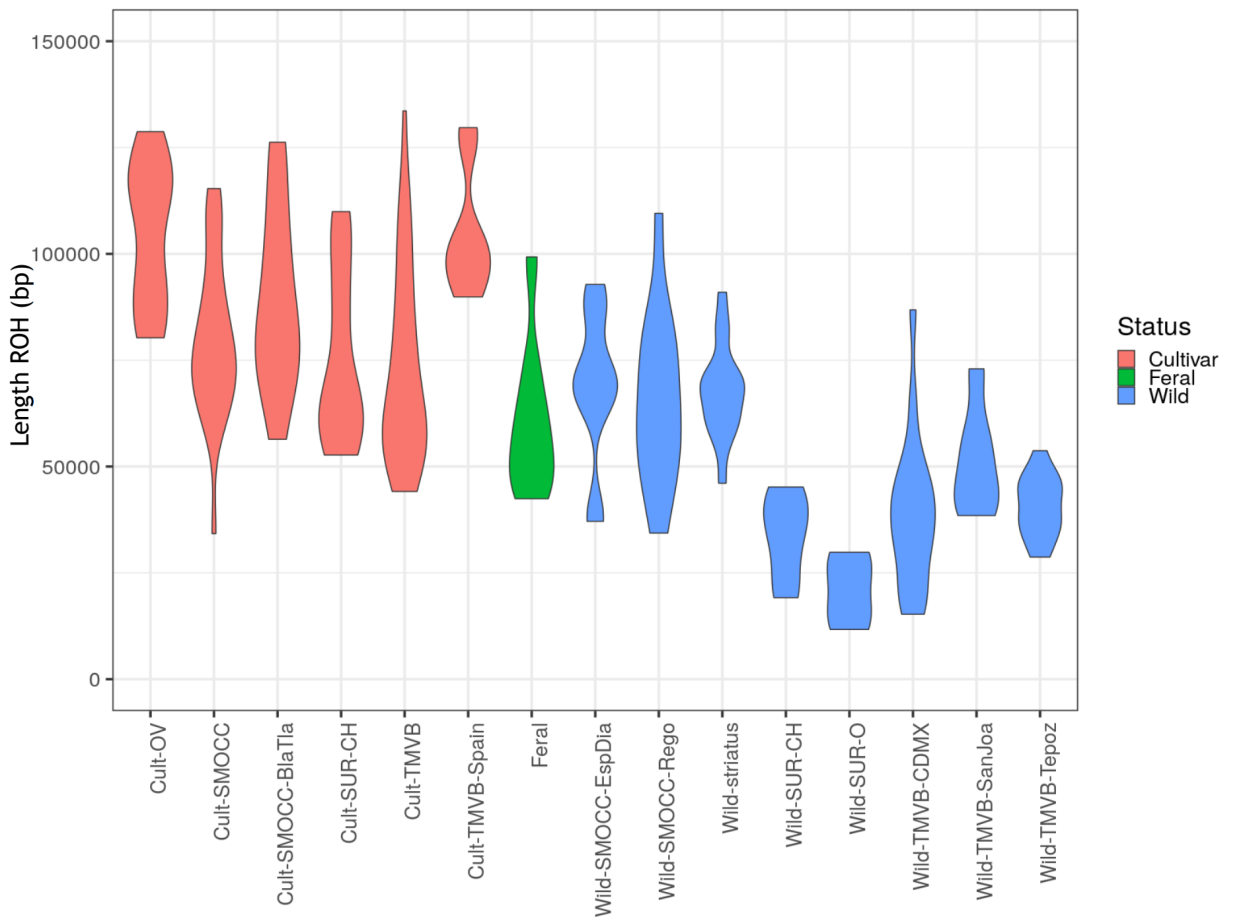


Fig. S9. Total length of ROH (Kb) estimated using a 500 Kb min window size. Colors indicate the type of *P. coccineus* sample.

**Table S6.** Demographic parameters estimated using fastsimcoal2 (95% CI). NWILD= *Ne* wilds; NCC= Current *Ne* cultivars; NAC= Ancestral *Ne* cultivars; NBOT= Ne during the bottleneck; TBOT= bottleneck time (generations); TEXP= time of demographic expansion; TDOM= domestication time; TDIV= divergence time; REXP= expansion rate; MIGWC= migration rate from wild to cult (NMWC/NWILD); MIGCW= migration rate from cult to wild (NMCW/NAC); NMWC= migrants from wild to cult; NMCW= migrants from cult to wild.

| **Pop-model** | **NWILD** | **NCC** | **NAC** | **NBOT** | **TBOT** | **TEXP** | **TDOM** | **TDIV** | **MIGWC** | **MIGCW** |
| --- | --- | --- | --- | --- | --- | --- | --- | --- | --- | --- |
| Cult-TMVB_Wild-  TMVB-constant | 425,777  (337,027 - 487,500) | 759,246  (716,234 - 834,945) | 2,423  (1,999 -  3,216) | NA | NA | 1,635  (1,522 - 1,775) | 9,701  (7,889 - 2,509) | NA | 1.38E-05  (1.14E-05 - 1.64E-05) | 6.19E-06  (2.66E-06 - 1.73E-05) |
| Cult-SMOCC_Wild-  SMOCC-recent | 355,636  (277,313 -  465,669) | 395,155  (273,532 - 622,906) | 17,574  (13,343 -  21,085) | NA | NA | 3,166  (3,006 - 3581) | NA | 162,039  (132,247 - 198,564) | 5.76E-06  (4.74E-06 - 7.07E-06) | NA |
| Cult-SUR_Wild-  SUR-CH-old | 459,744  (394,880 - 519,337) | 741,887  (558,292 -  921,143) | 8,571  (6,277 -  12,023) | NA | NA | 2,706  (2,167 - 2965) | NA | 176,689  (154,796 -  210,670) | 1.93E-05  (1.70E-05 - 2.20E-05) | NA |
| Cult-TMVB-Spain | NA | 4,130  (1,976 - 6,793) | 337,078  (241,184 - 495,681) | 1,047  (177 - 2,382) | 447  (156 -  753) | 247  (66 - 497) | NA | NA | NA | NA |
| Cult-OV | NA | 340,417  (325,384 - 355,451) | 218,142  (197,358 -  238,925) | 35,663  (33,873 - 37,453) | 11,116  (8,842 -  13,389) | 7,498  (6,217 - 8,780) | NA | NA | NA | NA |


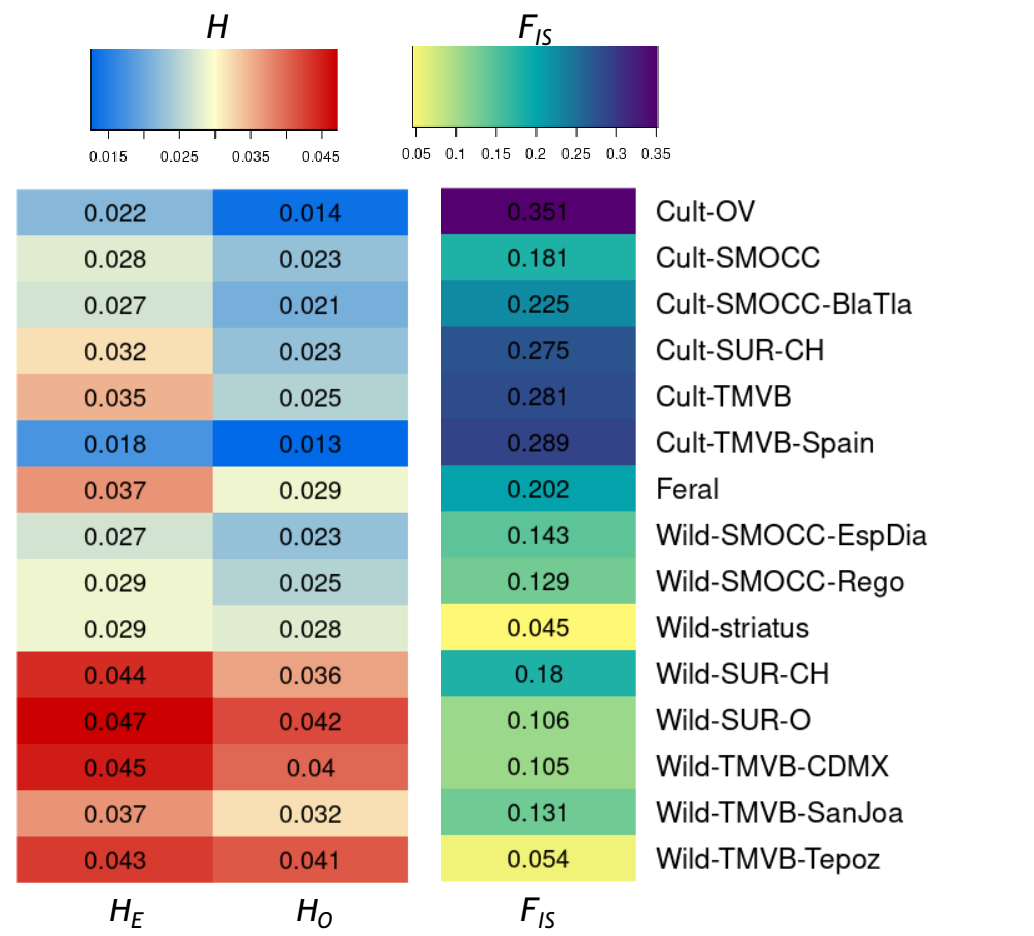


**Fig S10.** Heatmaps showing the genetic diversity in terms of *H* and inbreeding coefficient estimated for the 15 defined populations using the 237 samples.

**Table S7.** Genetic diversity levels in the *P. coccineus* populations. Expected (*H_E_*) and observed (*H_O_*) heterozygosity, and inbreeding coefficient (*F_IS_*). The confidence intervals were obtained performing 100 bootstraps. Asterisk shows the estimated values using the data subset (183 samples).

| **Population** | ***H_E_* (±SD)** | ***H_O_* (±SD)** | ***F_IS_*** | ***H_E_** (±SD)** | ***H_O_** (±SD)** | ***F_IS_**** |
| --- | --- | --- | --- | --- | --- | --- |
| Cult-OV | 0.022 (±0.008) | 0.014 (±0.004) | 0.351  (0.342-0.360) | 0.024 (±0.009) | 0.015 (±0.004) | 0.380  (0.371-0.388) |
| Cult-SMOCC | 0.028 (±0.009) | 0.023 (±0.006) | 0.181  (0.175-0.186) | 0.031 (±0.010) | 0.024 (±0.007) | 0.213  (0.207-0.220) |
| Cult-SMOCC-BlaTla | 0.027 (±0.009) | 0.021 (±0.006) | 0.225  (0.218-0.233) | 0.029 (±0.010) | 0.022 (±0.006) | 0.241  (0.233-0.249) |
| Cult-SUR-CH | 0.032 (±0.010) | 0.023 (±0.005) | 0.275  (0.270-0.279) | 0.035 (±0.011) | 0.024 (±0.006) | 0.304  (0.298-0.309) |
| Cult-TMVB | 0.035 (±0.010) | 0.025 (±0.005) | 0.281  (0.277-0.285) | 0.038 (±0.011) | 0.033 (±0.009) | 0.129  (0.125-0.134) |
| Cult-TMVB-Spain | 0.018 (±0.007) | 0.013 (±0.004) | 0.289  (0.276-0.302) | 0.020 (±0.008) | 0.014 (±0.005) | 0.289  (0.276-0.303) |
| Feral | 0.037 (±0.011) | 0.029 (±0.008) | 0.202  (0.195-0.208) | 0.042 (±0.012) | 0.032 (±0.008) | 0.242  (0.236-0.248) |
| Wild-SMOCC-EspDia | 0.027 (±0.008) | 0.023 (±0.006) | 0.143  (0.134-0.152) | 0.030 (±0.008) | 0.025 (±0.007) | 0.142  (0.133-0.150) |
| Wild-SMOCC-Rego | 0.029 (±0.008) | 0.025 (±0.007) | 0.129 (0.122-0.137) | 0.032 (±0.009) | 0.027 (±0.008) | 0.145  (0.138-0.151) |
| Wild-striatus | 0.029 (±0.010) | 0.028 (±0.010) | 0.045  (0.039-0.052) | 0.032 (±0.011) | 0.029 (±0.010) | 0.087  (0.080-0.094) |
| Wild-SUR-CH | 0.044 (±0.015) | 0.036 (±0.012) | 0.180  (0.171-0.189) | 0.048 (±0.016) | 0.039 (±0.014) | 0.187  (0.177-0.197) |
| Wild-SUR-O | 0.047 (±0.018) | 0.042 (±0.017) | 0.106  (0.096-0.117) | 0.052 (±0.020) | 0.046 (±0.019) | 0.108  (0.098-0.119) |
| Wild-TMVB-CDMX | 0.045 (±0.012) | 0.040 (±0.010) | 0.105  (0.101-0.110) | 0.050 (±0.013) | 0.042 (±0.010) | 0.154  (0.149-0.159) |
| Wild-TMVB-SanJoa | 0.037 (±0.013) | 0.032 (±0.011) | 0.131  (0.121-0.142) | 0.041 (±0.014) | 0.036 (±0.012) | 0.130  (0.119-0.141) |
| Wild-TMVB-Tepoz | 0.043 (±0.012) | 0.041 (±0.012) | 0.054  (0.048-0.060) | 0.048 (±0.013) | 0.045 (±0.013) | 0.058  (0.052-0.064) |


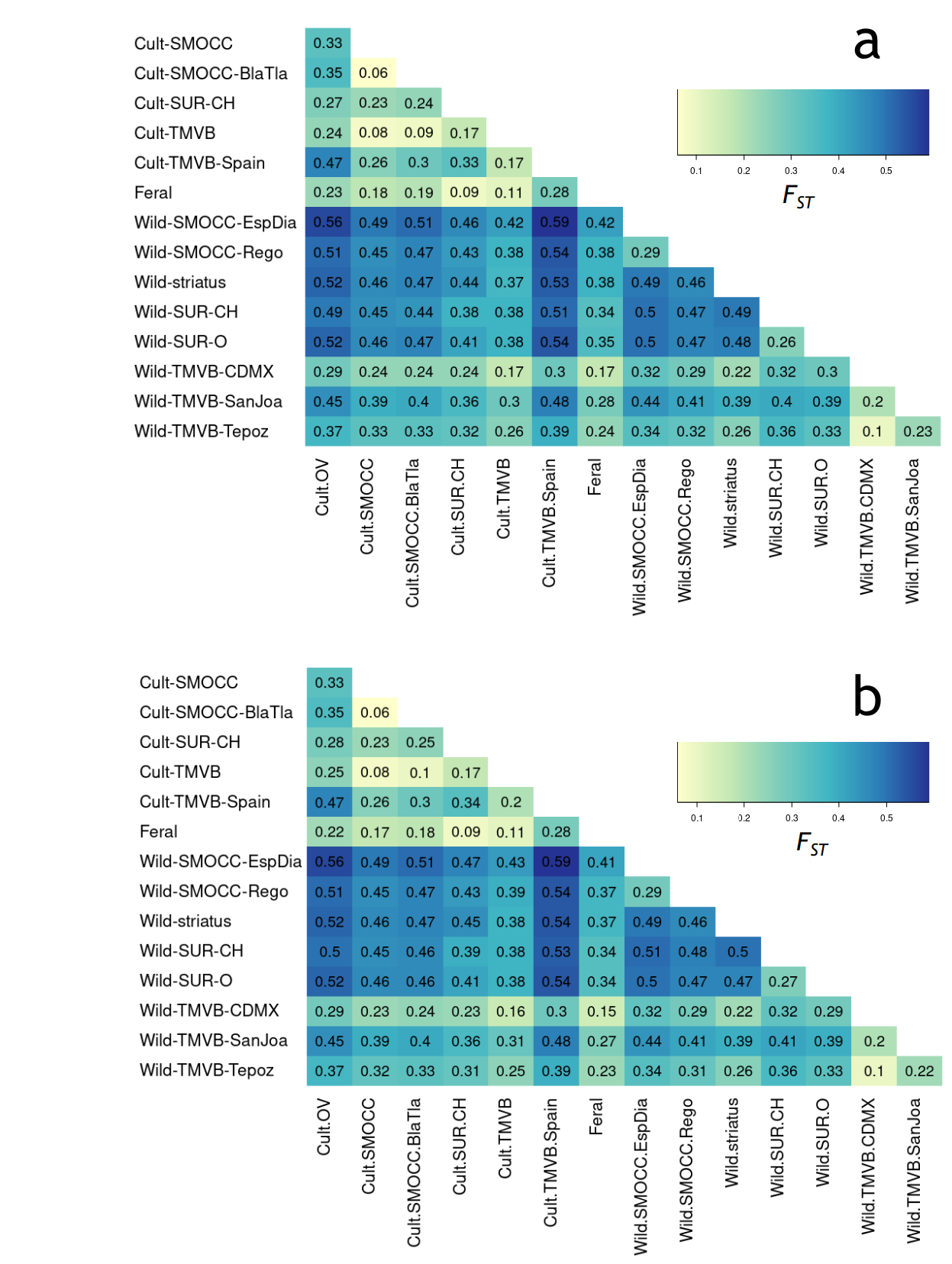


**Fig. S11.** Differentiation index among the 15 populations of *P. coccineus*. a) was estimated using the complete data set, and b) with the data subset (183 samples).


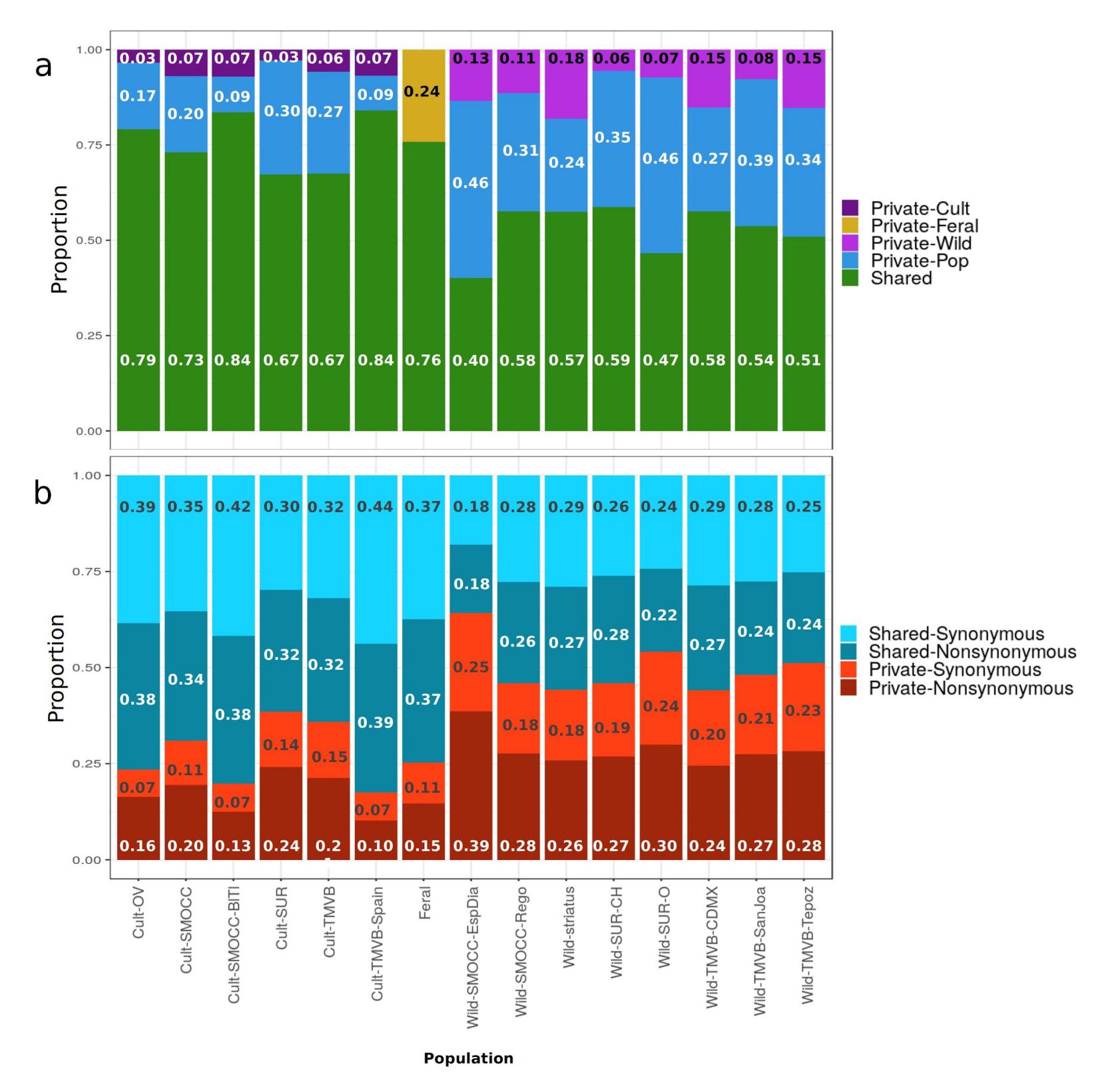


**Fig. S12.** a) Proportion of private and shares alleles for each population. b) Proportion of private and shared alleles within CDS regions, separating into synonymous and nonsynonymous mutations. The numbers inside the columns indicate the proportion of each category. Only segregating sites within populations are included.

**Table S8.** Proportion of segregating sites (SS) and nonsynonymous/synonymous ratio of SS splitted into the shared and private alleles found within each *P. coccineus* population.

| Population | Proportion | Nonsynonymous/synonymous | | |
| --- | --- | --- | --- | --- |
|  | SS | All SS | Shared SS | Private SS |
| Cult-OV | 0.080 | 1.196 | 0.991 | 2.306 |
| Cult-SMOCC | 0.140 | 1.138 | 0.953 | 1.708 |
| Cult-SMOCC-BlTl | 0.101 | 1.037 | 0.919 | 1.711 |
| Cult-SUR | 0.191 | 1.264 | 1.067 | 1.670 |
| Cult-TMVB | 0.237 | 1.148 | 1.008 | 1.452 |
| Cult-TMVB-Spain | 0.052 | 0.957 | 0.884 | 1.395 |
| Feral | 0.176 | 1.080 | 0.996 | 1.378 |
| Wild-SMOCC-EspDia | 0.134 | 1.294 | 0.986 | 1.511 |
| Wild-SMOCC-Rego | 0.138 | 1.174 | 0.948 | 1.519 |
| Wild-striatus | 0.102 | 1.113 | 0.928 | 1.405 |
| Wild-SUR-CH | 0.144 | 1.219 | 1.074 | 1.417 |
| Wild-SUR-O | 0.114 | 1.061 | 0.885 | 1.237 |
| Wild-TMVB-CDMX | 0.225 | 1.069 | 0.950 | 1.243 |
| Wild-TMVB-SanJoa | 0.117 | 1.067 | 0.876 | 1.320 |
| Wild-TMVB-Tepoz | 0.186 | 1.078 | 0.937 | 1.234 |


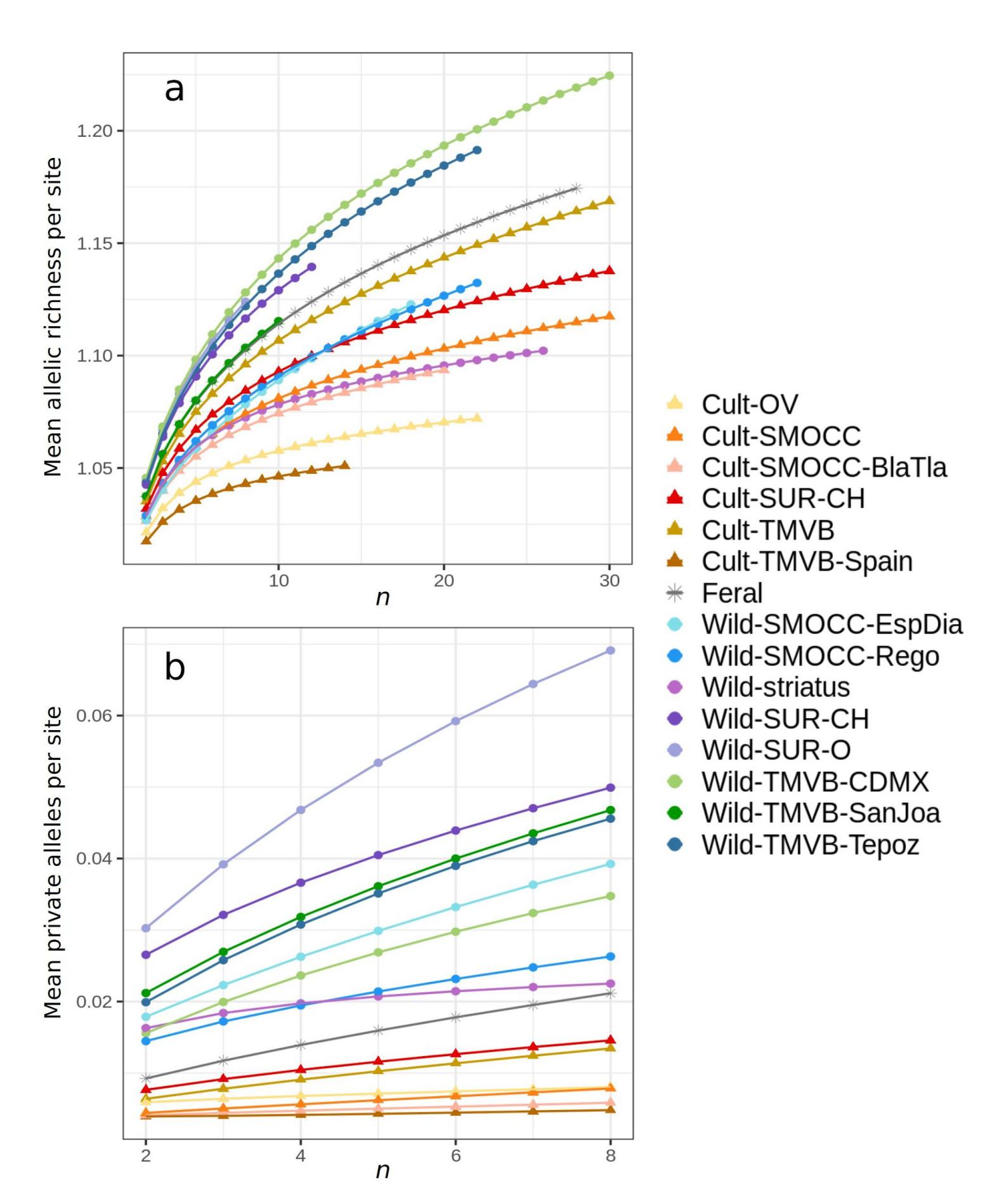


Fig. S13. A) Mean allelic richness per site and (B) mean private alleles per site estimated with AZDE for each population.


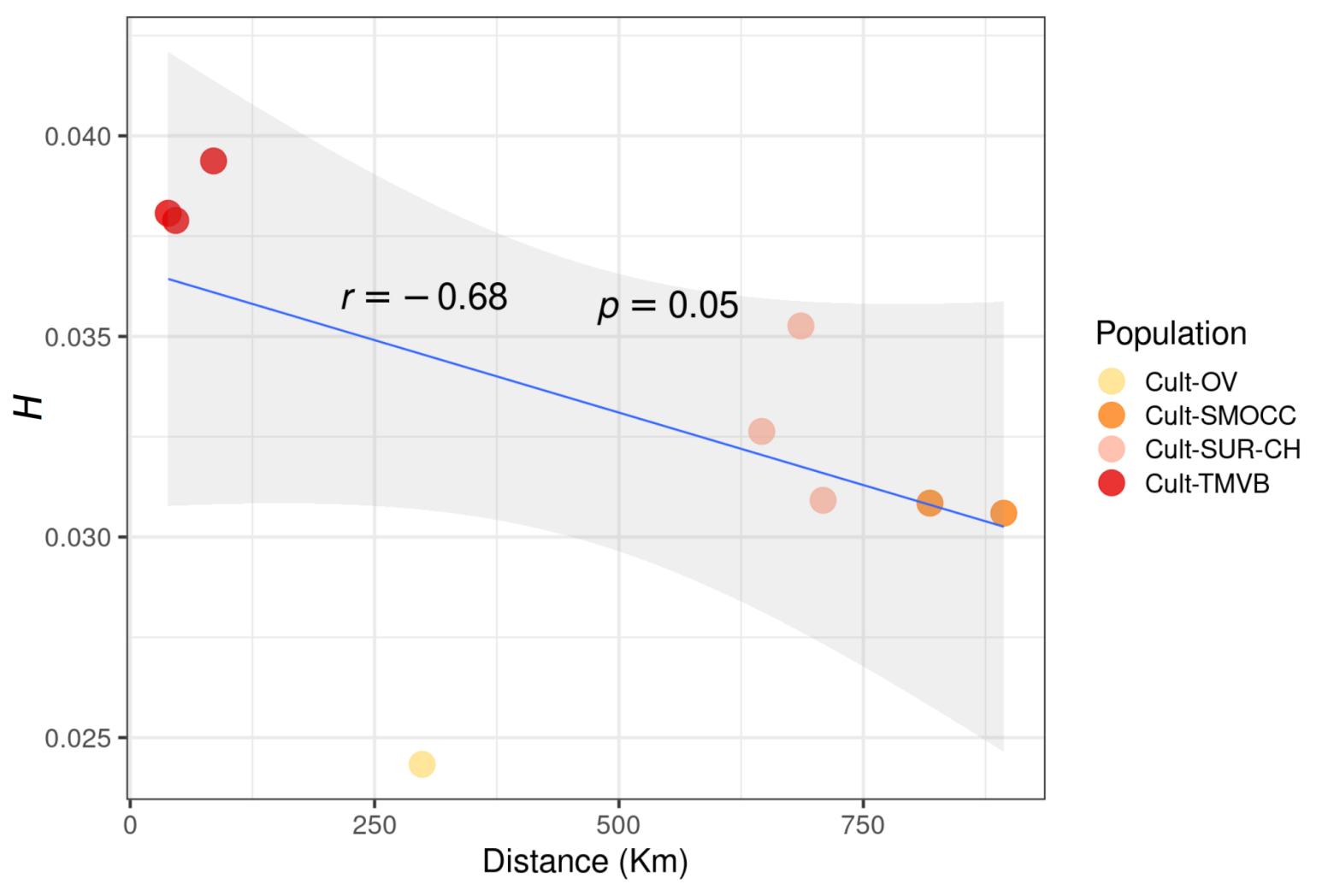


Fig. S14. Correlation between the genetic diversity (*H_E_*) and the distance from the centroid of the Cult-TMVB locations to traditional variety locations estimated using the data subset (183 samples).
